# Osteological profiling of femoral diaphysis and neck in aquatic, semiaquatic, and terrestrial carnivores and rodents: effects of body size and locomotor habits

**DOI:** 10.1007/s00360-024-01551-7

**Published:** 2024-04-27

**Authors:** Petteri Nieminen, Mikko A. J. Finnilä, Wilhelmiina Hämäläinen, Saara Lehtiniemi, Timo Jämsä, Juha Tuukkanen, Mervi Kunnasranta, Heikki Henttonen, Anne-Mari Mustonen

**Affiliations:** 1 Department of Environmental and Biological Sciences, Faculty of Science, Forestry and Technology, University of Eastern Finland, Joensuu, Finland; 2 School of Medicine, Institute of Biomedicine, Faculty of Health Sciences, University of Eastern Finland, Kuopio, Finland; 3 Research Unit of Health Sciences and Technology, Faculty of Medicine, University of Oulu, Oulu, Finland; 4 Biocenter Oulu, University of Oulu, Oulu, Finland; 5 Department of Computer Science, School of Science, Aalto University, Espoo, Finland; 6 Research Unit of Translational Medicine, Department of Anatomy and Cell Biology, Faculty of Medicine, University of Oulu, Oulu, Finland; 7 Natural Resources Institute Finland, Joensuu, Finland; 8 Natural Resources Institute Finland, Vantaa, Finland

**Keywords:** Aquatic mammals, Bone mineral density, Bone strength, Lifestyle, Semiaquatic mammals, Terrestrial mammals

## Abstract

The increased limb bone density documented previously for aquatic tetrapods has been proposed to be an adaptation to overcome buoyancy during swimming and diving. It can be achieved by increasing the amount of bone deposition or by reducing the amount of bone resorption, leading to cortical thickening, loss of medullary cavity, and compaction of trabecular bone. The present study examined the effects of locomotor habit, body size, and phylogeny on the densitometric, cross-sectional, and biomechanical traits of femoral diaphysis and neck in terrestrial, semiaquatic, and aquatic carnivores, and in terrestrial and semiaquatic rodents (12 species) by using peripheral quantitative computed tomography, three-point bending, and femoral neck loading tests. Groupwise differences were analyzed with the univariate generalized linear model and the multivariate linear discriminant analysis supplemented with hierarchical clustering. While none of the individual features could separate the lifestyles or species adequately, the combinations of multiple features produced very good or excellent classifications and clusterings. In the phocid seals, the aquatic niche allowed for lower femoral bone mineral densities than expected based on the body mass alone. The semiaquatic mammals mostly had high bone mineral densities compared to the terrestrial species, which could be considered an adaptation to overcome buoyancy during swimming and shallow diving. Generally, it seems that different osteological properties at the levels of mineral density and biomechanics could be compatible with the adaptation to aquatic, semiaquatic, or terrestrial niches.

**Supplementary Information:**

The online version contains supplementary material available at 10.1007/s00360-024-01551-7.

## Introduction

Morphological specializations in the locomotor system have likely played a fundamental role in mammalian evolution, and the lengths, diameters, and proportions of limb bones vary strongly with locomotor habits (Kilbourne 2017; Kilbourne and Hutchinson 2019). The adaptations required for the transition from the terrestrial to the aquatic lifestyle include the streamlined body form, propulsive appendages, and internal and external structural alterations for buoyancy control (Fish and Stein 1991). Relative increase in body mass (BM) is also present in semiaquatic and fully aquatic mammals (Farina et al. 2023). Higher limb bone densities have been documented in some aquatic tetrapods compared to their terrestrial relatives, a phenomenon considered to be an adaptation to overcome buoyancy during swimming and diving and achieved by increasing the amount of bone deposition or by reducing resorption (Wall 1983; Fish and Stein 1991; Gray et al. 2007; Houssaye 2009; Nakajima and Endo 2013). This leads to cortical thickening, loss of medullary cavity, and compaction of trabecular bone. Pachyostosis corresponds to the hyperplasia of periosteal cortices leading to increased volume, while osteosclerosis is defined as an increase of bone inner compactness with no effect on the external morphology (Houssaye 2009). Some highly aquatic deep-diving marine mammals, such as cetaceans and elephant seals (*Mirounga* spp.), have been proposed to display secondarily reduced bone density (Wall 1983; Gray et al. 2007). These species are capable of lung collapse during deep dives and, for this reason, would not need an elevated skeletal mass.

Semiaquatic species occupy an intermediate position between terrestrial and aquatic animals, as they are not specialized for either environment (Fish 2000). They can be considered modern analogs of evolutionary intermediates between ancestral terrestrial mammals and their fully aquatic descendants. Semiaquatic mammals require two different modes of locomotion as they move through both water and air with different densities and viscosities (Botton-Divet et al. 2016, 2017). Gravity is the main external force affecting terrestrial locomotion, whereas moving through the aquatic environment involves important drag-based forces that form the principal constraints on locomotion, when gravity is offset by buoyancy. The semiaquatic taxa do not occupy a clearly distinct ecological category, but there is a lot of variation depending on the swimming mode, swimming depth, degree of dependence on the terrestrial environment, and efficiency of terrestrial locomotion. Semiaquatic species typically use non-wettable fur to control their buoyancy while blubber, a thick layer of vascularized adipose tissue under the skin, is utilized by fully aquatic mammals (Fish 2000; Fish et al. 2002), such as cetaceans, but also by pinnipeds that spend a considerable amount of time on land but forage in the aquatic environment (Reidenberg 2007). The locomotor system of semiaquatic species reflects a trade-off between the different functional constraints it faces (Botton-Divet et al. 2016). They have only limited morphological and microanatomical adaptations to life in water due to the demands of terrestrial locomotion, and do not usually display profound osteological modifications (Stein 1989; Houssaye 2009).

The shape and microanatomy of long bones have been previously compared between (semi)aquatic mammals and their terrestrial relatives (Stein 1989; Fish and Stein 1991; Nakajima and Endo 2013; Botton-Divet et al. 2016, 2017). Recent studies have focused especially on the cross-sectional properties and microanatomy of mustelid limb bones (Houssaye and Botton-Divet 2018; Amson and Kilbourne 2019; Kilbourne and Hutchinson 2019) but, to the best of our knowledge, detailed bone biomechanical properties have not been studied in this respect. The aim of the present study was to examine the densitometric, cross-sectional, and biomechanical traits of femoral diaphysis and neck in reference to aquatic, semiaquatic, and terrestrial lifestyles and between-species differences, taking into account the potential effect of BM. In addition, we assessed if the clade of the sampled species (Carnivora and Rodentia) would show effects different from niche on the osteological properties. These were realized by using peripheral computed tomography (pQCT), three-point bending, and femoral neck loading tests, to gain an up-to-date understanding of the osteology in several mammalian species of different niches. It was hypothesized that especially aquatic but, to some degree also semiaquatic mammals, would have elevated bone densities to overcome buoyancy during swimming and diving.

## Methods

### Origin of specimens

The specimens examined in this study consisted of 7 carnivore and 5 rodent species that were laboratory-bred, farm-reared, or wild animals (Table 1) and originated from Finland (61º–68º N). The species were selected based on their lifestyle (aquatic, semiaquatic, or terrestrial), locomotor specializations for moving on land and through water, and availability of specimens. The farm-reared and laboratory-bred animals were of known age, while the wild animals were classified into juveniles and adults based on the histological examination of teeth and/or the epiphyseal closure of long bones. Both male and female specimens were examined when available.

**Table 1:** Specimens examined in the study

Species	Common name	Habitat	Origin	N	Sex	Age	Body mass, g^a^
*Halichoerus grypus*	Baltic gray seal	Aquatic	Wild	9	3 M, 6F	4 J, 5A	93,556 ± 14,791
*Phoca/Pusa hispida botnica*	Baltic ringed seal	Aquatic	Wild	9	6 M, 3F	5 J, 4A	32,625 ± 5186
*Lutra lutra*	Eurasian otter	Semiaquatic	Wild	22	13 M, 8F, 1 nd	nd	5897 ± 381
*Neovison vison*	American mink	Semiaquatic	Farm-reared	12	6 M, 6F	12 J	2080 ± 208
*Mustela putorius*	European polecat	Terrestrial	Farm-reared	16	16 M	16A	2016 ± 40
*Martes zibellina*	Sable	Scansorial	Farm-reared	16	8 M, 8F	16 J	1177 ± 45
*Nyctereutes procyonoides*	Raccoon dog	Terrestrial	Wild	51	26 M, 25F	2A, 48 J, 1 nd	5873 ± 182
*Ondatra zibethicus*	Muskrat	Semiaquatic	Wild	7	5 M, 2F	1 J, 6A	1287 ± 68
*Arvicola amphibius*	Water vole	Semiaquatic	Wild	13	8 M, 5F	6 J, 7A	204 ± 12
*Myodes glareolus*	Bank vole	Terrestrial	Wild	28	16 M, 12F	21 J, 7A	23 ± 0.6
*Microtus oeconomus*	Tundra vole	Terrestrial	Laboratory-bred	30	15 M, 15F	28 J, 2A	25 ± 1.0
*Microtus arvalis*	Common vole	Terrestrial	Laboratory-bred	30	15 M, 15F	30 J	22 ± 1.0

^a^ = mean ± SE, M = male, F = female, A = adult, J = juvenile, nd = not determined

Baltic grey (*Halichoerus grypus*) and ringed seal (*Phoca hispida botnica*) samples were collected in the Bothnian Bay under the permit from the Ministry of Agriculture and Forestry (1121/722/2008) for scientific sampling to study the dietary habits and toxin load of the species. Laboratory-bred tundra (*Microtus oeconomus* or *Alexandromys oeconomus*) and common voles (*M. arvalis*) were sampled by the permission of the Finnish National Animal Experiment Board (ESLH-2007-06169/Ym-23). According to the Finnish Act on the Use of Animals for Experimental Purposes (62/2006) and a further decision by the National Animal Experiment Board (16 May, 2007), the live-trapping of wild bank voles (*Myodes glareolus* or *Clethrionomys glareolus*) was not an animal experiment. No permit was required to euthanize and subsequently sample the bank voles (Finlex Data Bank 2023). Nor was any animal ethics license required for the sampling of the American mink (*Neovison vison*), European polecats (*Mustela putorius*), and sables (*Martes zibellina*) that were farmed commercially until they were euthanized according to the recommended practices (Council of the European Union 2009) during the pelting season. Wild raccoon dogs (*Nyctereutes procyonoides*) and muskrats (*Ondatra zibethicus*) were obtained as fresh carcasses. Both species are invasive in Finland and legally hunted year-round. The trapping of wild water voles (*Arvicola amphibius*) with killing snap traps was allowed by the Finnish hunting legislation without special permits (Finlex Data Bank 2023). Wild Eurasian otters (*Lutra lutra*) were obtained as fresh carcasses of individuals found dead and delivered to the zoological collection of the former University of Joensuu, Finland.

### Osteological measurements

The femur was the bone of choice for osteological measurements as it is of adequate size even in small species and important for locomotion, albeit for different purposes on land and in water (de Rudolf 1922). The left femur, when available, was detached whole and preserved within the surrounding tissues at −40 to −80 °C, otherwise the right femur was studied. The femurs were subsequently cleansed and measured for mass and dimensions with a digital caliper and stored in phosphate buffered saline until analysis. The femoral volume was determined by submerging the bone and by measuring the volume of water displaced.

The bone mineral density (MD) and cross-sectional morphology at the mid-shaft of femoral diaphysis and at femoral neck were examined with pQCT using the Stratec XCT 960A instrument with the *v*5.20 software (Norland Stratec Medizintechnik GmbH, Birkenfeld, Germany) at the Faculty of Medicine, University of Oulu, Finland (see details in Nieminen et al. 2011; Mustonen et al. 2017). The resolution of 0.092 mm × 0.092 mm to 0.689 mm × 0.689 mm with a constant slice depth of 1.25 mm was utilized (Supplementary Table S1). After the pQCT measurements, the mechanical properties of the femurs were tested with the three-point bending and femoral neck loading tests (Instron 3366, Norwood, MA, USA), as described previously (Nieminen et al. 2011; Mustonen et al. 2017). A total of 6 pQCT variables and 8 biomechanical variables were determined for the femoral diaphysis, and 10 pQCT variables and 8 biomechanical variables for the femoral neck (Table 2).

**Table 2:** Measured osteological variables

Variable	Unit
Total/cortical/trabecular mineral density	mg/cm^3^
Total/cortical/trabecular mineral content	mg
Total/cortical/trabecular area	mm^2^
Cortical thickness	mm
Periosteal/endosteal circumference	mm
Stiffness (slope of the linear portion of the load–deformation curve)	N/mm
Deformation at yield (amount of displacement at yield point between elastic and plastic regions)	mm
Force at yield (load at yield point between elastic and plastic regions)	N
Energy at yield (area under the load–deformation curve up to yield point)	mJ
Deformation at maximum load (amount of displacement at maximum load point)	mm
Force at maximum load (maximum value of load attained during the test)	N
Energy at maximum load (area under the curve up to maximum load point)	mJ
Toughness (area under the stress–strain curve up to the point of failure)	mJ

For some species, all variables could not be measured due to certain species-specific bone characteristics. The femoral necks of the seals were too short and robust (Adam 2008) to be tested with the same femoral loading test used for the other species, and those of *Myodes* and *Microtus* voles were too tiny for the pQCT measurements. Parts of the raccoon dog and bank vole data, measured as described above, were previously published (Nieminen et al. 2011, 2015).

### Statistical evaluation of individual features and their relationships

The statistical evaluation was performed with the IBM SPSS Statistics (*v*25 software, IBM, Armonk, NY, USA). In all tests, individuals with missing values in the studied variables were excluded, and the threshold *α* = 0.05 was used to determine statistically significant discoveries.

First, all features were analyzed separately to evaluate differences between species, the effect of animal size, and whether the effect depended on the lifestyle (aquatic, semiaquatic, or terrestrial). Basic statistics (mean ± SE) were calculated for femoral mass, volume, and dimensions for each species. The logarithmic relationship between femoral volume and mass was studied and, to examine if the bones of larger mammals were stronger than those of smaller species, correlations between the strength (force at maximum load, F_max_) and size variables were analyzed after *x*^*2*^ and* x*^*2/3*^ transformations (Currey 2002). Spearman correlation coefficient (r_s_) was used to evaluate monotonic dependencies, since it was expected that the relationships were non-linear. Allometric equations for particular osteological variables and BM were determined based on Moore (2000). The variables were plotted after log-transformation on a scatter plot and the equations of the regression lines were converted to conventional allometric equations of the form y = bx^m^ with standard mathematical operations.

Species-wise basic statistics were also calculated for all pQCT and biomechanical variables of the femoral diaphysis and neck, and for their BM-normalized versions that have also been calculated in previous studies (Zernicke et al. 1995; LaMothe et al. 2003; Sornay-Rendu et al. 2013). The effect of BM on each variable and possible lifestyle interaction were evaluated with the univariate generalized linear model (GLM) using the examined variable as a dependent (response) variable, BM as a covariate, lifestyle as a factor, lifestyle × BM as an interaction term, and assuming normal distribution of the response variable.

### Linear discriminant analysis

Linear discriminant analysis (LDA) was used to analyze how well the pQCT and biomechanical variables could separate the different lifestyles (divided into 3 classes: aquatic, semiaquatic, and terrestrial) or the species (9 or 6 classes, depending on the data). Altogether, 14 LDA models were learnt for different classification tasks.

In the analysis of the femoral diaphysis data, all pQCT and three-point bending test variables were used but, due to missing data, *N. procyonoides* and *Microtus* voles were excluded as well as all individuals with missing values. The dataset contained 9 species from all 3 lifestyles (n = 121). For comparison, both lifestyle and species classifiers were learnt using either the original variables or their BM-normalized versions, together with cortical MD, for which the BM correction was not meaningful.

In the case of the femoral neck data, all variables could not be used to learn a three-class lifestyle classifier since the seals (the only aquatic animals) were missing the loading test variables. Therefore, two alternative models were analyzed: first, a three-class model (aquatic, semiaquatic, or terrestrial) using all pQCT variables and 8 species (n = 87), excluding *N. procyonoides* and *Myodes* and *Microtus* voles, and second, a two-class model (semiaquatic or terrestrial) using the femoral neck loading test variables and 6 species (n = 66), excluding seals, *N. procyonoides*, and *Myodes* and *Microtus* voles. In the species classification, all femoral neck variables were used to classify the above-mentioned 6 species. All models were constructed using either the original variables or their BM-normalized versions, accompanied by total and trabecular MDs, for which the normalization was not meaningful.

In the final LDA experiment, extra models were constructed to separate the American mink and European polecat, as examples of species with a similar BM and body shape but different lifestyles (n = 27 in the diaphysis data and n = 25 in the neck data after removing individuals with missing values). Four classifiers were constructed, using either the original or the BM-normalized variables together with MD variables, both for the diaphysis and femoral neck data.

The accuracy of classifiers was evaluated with leave-one-out (LOO) cross validation. In addition, the most important features for each model were determined from the loadings (weights) of variables in the first discriminant, which explains most of between-class variance. The optimal transformation of data for lifestyle classification was also presented visually as 2-dimensional scatterplots along the first linear discriminants, showing the classes of individual data points with different symbols. The modelling was implemented with Python using the Linear discriminant analysis package of Scikit-learn 1.2.0 (Pedregosa et al. 2011).

### Hierarchical clustering

Hierarchical clustering (HC) was used to analyze natural groupings and group structures in the pQCT and biomechanical data and to evaluate how well they matched the species and lifestyle classifications. HC was selected, because it produces a visual presentation of the cluster hierarchy as a dendrogram, a tree structure, whose bottom level corresponds to individual data points (singleton clusters), the branches show how clusters were combined, and the height of each branch reflects the distance between corresponding subclusters. A partition of data into K subgroups corresponds to cutting the hierarchy at a suitable level. The clustering was performed in an agglomerative (bottom-up) manner, proceeding from individual data points to larger clusters.

The main differences between HC methods stem from different linkage metrics that determine the distances between clusters. Therefore, we compared five commonly used linkage metrics: single-link (distance between the closest points of two clusters C_1_ and C_2_), complete-link (distance between the farthest points in C_1_ and C_2_), average-link (average distance between all points in C_1_ and C_2_), distance between centroids (the center-most points) of C_1_ and C_2_, and the Wardʼs method that evaluates the difference in the sum of squared errors (distances from centroids), SSE, when C_1_ and C_2_ are combined [*i.e.*, SSE(C_1_ $\cup$ C_2_) − SSE(C_1_) − SSE(C_2_)]. Distances between individual data points were measured with the Euclidean distance.

Results of HC depend heavily on the features that are used to present the data, and they cannot contain any missing values. In the diaphysis data, there were 11 features that were measured from all 12 species, while in the neck data, no features were measured from all species, but there were 9 features that were measured from 8 species (excluding *Myodes* and *Microtus* voles and *N. procyonoides*). All individuals with missing values in these features were removed, which left n = 232 in the diaphysis dataset and n = 95 in the neck dataset. All features were scaled to interval [0,1] using min–max scaling. Optimal features were determined with an iterative greedy search, by dropping features one by one from the original 11 (diaphysis) or 9 (neck) features as long as the clustering quality improved.

The clustering quality, *i.e.*, selection of optimal features, linkage metric, and number of clusters (K), was evaluated using normalized mutual information (NMI) by Strehl and Ghosh (2002):

$${\text{NMI}}\, = \,\frac{MI(C,D)}{{\sqrt {H(C)} \sqrt {H(D)} }},$$

where C is the set of clusters and D is the set of classes. NMI estimates mutual information (MI) between a clustering and a given classification (species division), normalized by the geometric mean of their entropies (H). NMI obtains values in [0,1], where high values indicate strong correspondence between the clustering and classification. For comparison, optimal clusterings were also determined for both datasets with the same features when lifestyle (aquatic, semiaquatic, or terrestrial) was used as a reference classification in NMI calculation.

The entire clustering hierarchies from individual animals to one overall cluster were presented visually as dendrograms, showing the classes of animals with different colours. For the interpretation of dendrograms, the mean values and ranges of variables were compared between clusters to find out if the cluster division was solely due to one or a few variables. In addition, the contents of optimal clusterings (with best NMIs) were also presented as confusion matrices that show the distribution of individuals in different classes (rows) and clusters (columns). From the confusion matrices, one can easily evaluate which classes get confused (members fall into the same clusters), how pure the clusters are (ideally, only one dominating class per cluster), and if the class is heterogenous (members fall into multiple clusters), with the given distance function and feature combination. The clustering was implemented with Python using the Cluster package of Scikit-learn 1.2.1 (Pedregosa et al. 2011).

## Results

### Statistical evaluation of individual features and their relationships

**Fig. 1:**
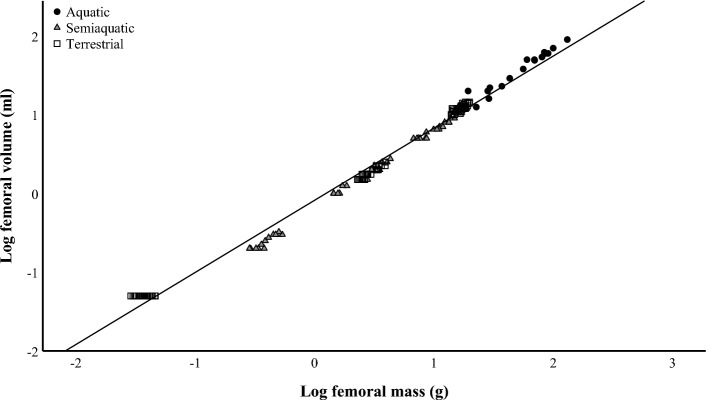
Relationships between log femoral mass and log femoral volume in the studied aquatic, semiaquatic, and terrestrial mammals

**Fig. 2:**
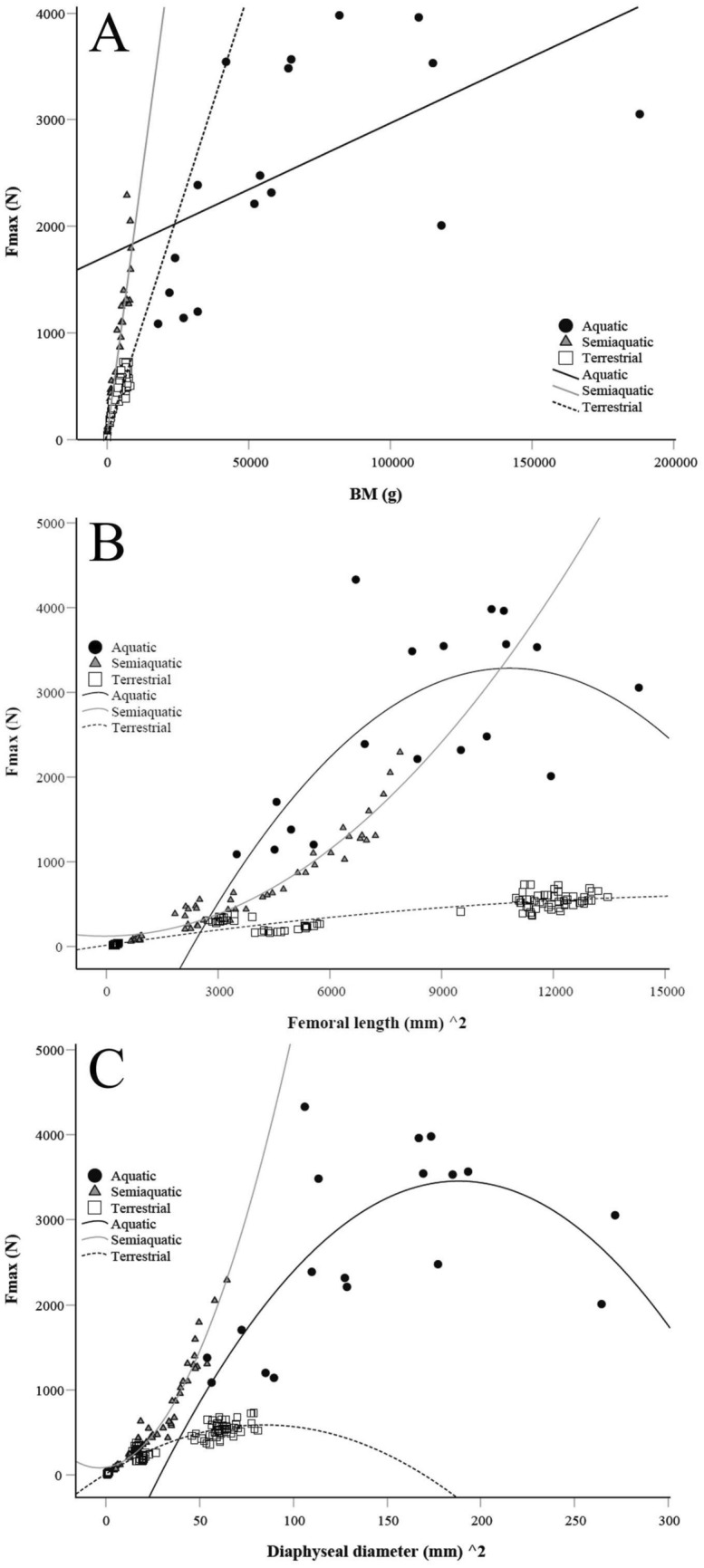
Relationships between the force at maximum load (F_max_), body mass (BM), femoral length, and diameter of femoral diaphysis in aquatic, semiaquatic, and terrestrial mammals. **a** Aquatic: r_s_ = 0.687, *p* = 0.002; semiaquatic: r_s_ = 0.946, *p* < 0.001; terrestrial: r_s_ = 0.919, *p* < 0.001; **b** aquatic: r_s_ = 0.583, *p* = 0.011; semiaquatic: r_s_ = 0.957, *p* < 0.001; terrestrial: r_s_ = 0.919, *p* < 0.001; **c** aquatic: r_s_ = 0.552, *p* = 0.018; semiaquatic: r_s_ = 0.979, *p* < 0.001; terrestrial: r_s_ = 0.949, *p* < 0.001

Basic statistics of femoral morphology (mass, volume, and dimensions) for each species are reported in Supplementary Table S2 with obvious differences in mass and size because of the wide range of BMs in the studied species. The relationship between femoral mass and volume was logarithmic, as shown in Fig. 1. The F_max_ of diaphysis correlated positively with BM, squared femoral length, and squared diaphyseal diameter in all lifestyle groups, albeit the spread of values was higher within the seals (Fig. 2), verifying that the larger mammals had higher absolute bone strength values compared to the smaller species. In all ecological niches, F_max_ showed negative allometry with BM, most clearly in the aquatic seals (Supplementary Fig. S1A).

**Fig. 3:**
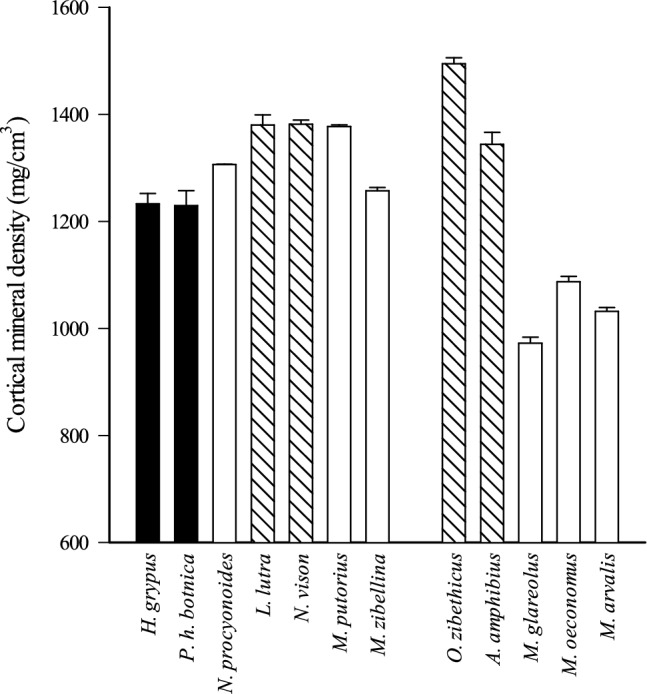
Cortical mineral density in femoral diaphysis of the studied carnivores and rodents (mean + SE). Black bars = aquatic species, striped bars = semiaquatic species, white bars = terrestrial species

Basic statistics of the pQCT and three-point bending variables of the femoral diaphysis are presented in Supplementary Tables S3 and S5 (original variables) and S7 and S9 (BM-normalized variables). The aquatic seals had the highest mean values in most original variables, but cortical MD was lower in comparison to semiaquatic species and most terrestrial carnivores (Fig. 3). The semiaquatic species had high cortical MDs, and especially *O. zibethicus* showed high values in many pQCT parameters and in stiffness and F_max_ when compared to other species with similar or higher BMs. In the univariate GLM, all diaphysis variables showed a positive association with BM and lifestyle × BM interaction. After BM-normalization, the seals had lower mean values in most variables compared to the terrestrial counterparts, and *Myodes* and *Microtus* voles often showed the highest averages.

Basic statistics of the pQCT variables of the femoral neck are presented in Supplementary Tables S4 (original variables) and S8 (BM-normalized). The aquatic species showed the highest mean values in most original variables, but the total and trabecular MDs were lower in comparison to many other species, with the lowest densities in *H. grypus*. In addition, *O. zibethicus* and *A. amphibius* showed higher total and trabecular MDs. Most of the pQCT variables of the femoral neck showed a positive relationship with BM, but for total and trabecular MDs the relationship was negative. Lifestyle × BM interaction was found for all measured variables. After BM-normalization, the seals had lower and the semiaquatic rodents higher average values in most variables compared to the terrestrial species.

Basic statistics of the neck loading test variables are presented in Supplementary Tables S6 (original variables) and S10 (BM-normalized). The seals did not provide data from the neck loading tests, as their bone shape was not suitable for the method. Regarding the original measurements, *L. lutra* showed low stiffness but high values in other variables, especially in energy at yield (E_yield_), energy at maximum load (E_max_), and toughness. Most of the femoral neck loading variables showed a positive relationship with BM, and there was lifestyle × BM interaction for F_max_ and toughness. After BM-normalization, *M. glareolus* and *A. amphibius* had higher mean values in several variables, but *A. amphibius* showed the lowest values in E_max_ and toughness.

### Linear discriminant analysis

**Fig. 4:**
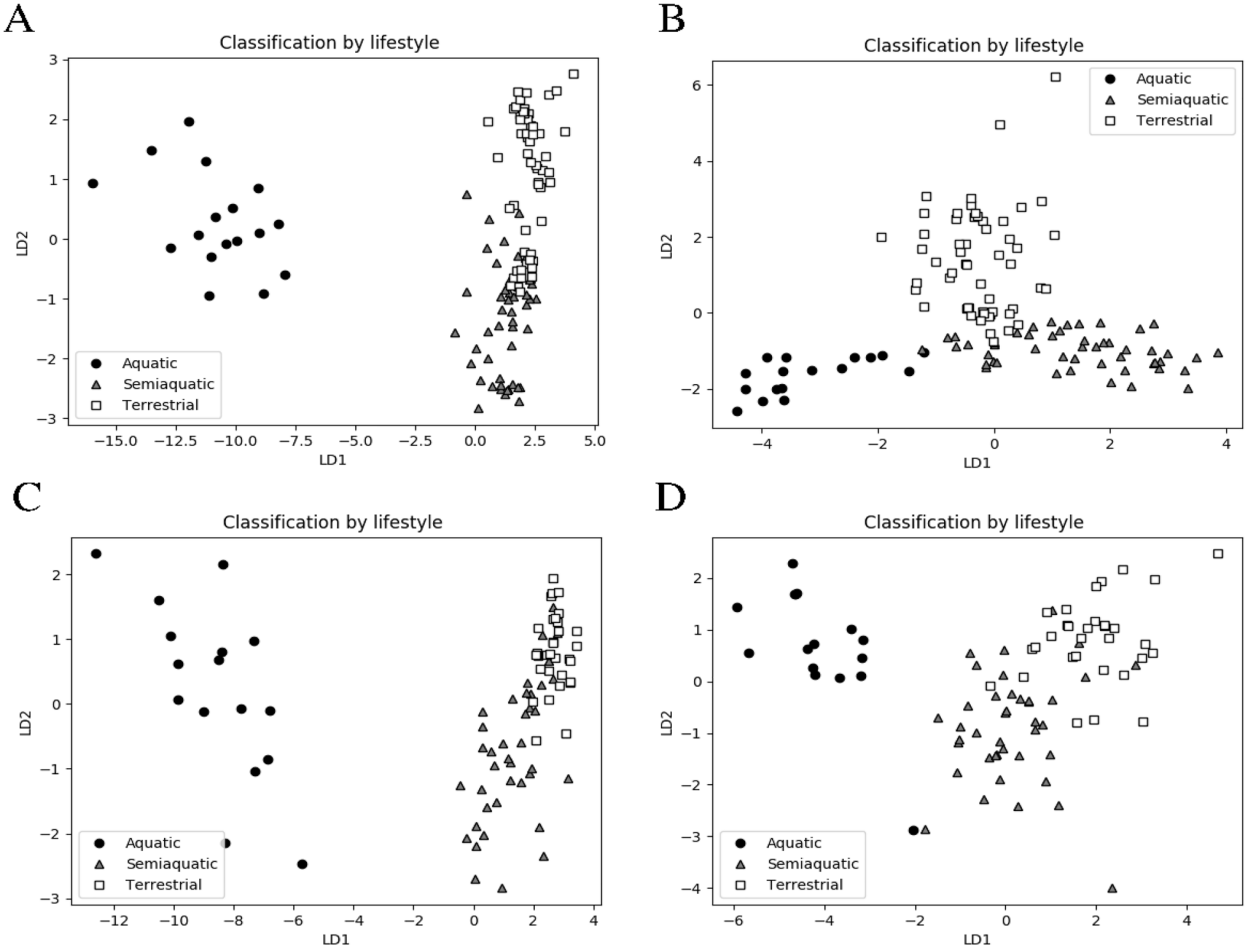
Scatter plots of femoral diaphysis and neck data along the linear discriminants LD1 and LD2. The linear discriminant analysis models were constructed for lifestyle classification using unnormalized (**a**) and body mass (BM)-normalized (**b**) pQCT and three-point bending variables of the femoral diaphysis, and unnormalized (**c**) and BM-normalized (**d**) pQCT variables of the femoral neck. Black symbols = aquatic species, grey symbols = semiaquatic species, white symbols = terrestrial species

In the femoral diaphysis data, the classification accuracy of the LDA models evaluated by the LOO cross validation was very good or even excellent. The 3 lifestyles could be classified with 84.3% accuracy using the original pQCT and bending test variables and with 90.1% accuracy using their BM-normalized versions. The aquatic class was well separated from the other two classes; with the original features the precision and sensitivity were 100%, and after BM-normalization, both were 94.1%. The most important features were cortical area and cortical mineral content (MC), irrespective of the BM-normalization, but also periosteal circumference, E_max_, and toughness were important among unnormalized features. The scatter plots of the data along the linear discriminants (Fig. 4a, b) show that the aquatic species are well separated from the other groups, but there is some overlap between the semiaquatic and terrestrial groups. In Fig. 4b, one can also observe a single semiaquatic data point almost overlapping an aquatic one, both of which were classified as aquatic by the LDA model. This represents a *N. vison* individual with the lowest values in cortical MD and BM-normalized cortical MC and stiffness for the species, while the overlapping *P. hispida botnica* individual has the highest values in the same features among all seals. These explain the misclassification, since cortical MD and stiffness had more weight in the BM-normalized LDA model.

At the species level (9 species), the classification accuracy was even better, 96.7% with the original features and 90.1% with their BM-normalized equivalents. Most species were detected perfectly, except *N. vison vs. M. putorius*, the 2 seal species, and also a few misclassified *L. lutra* in the latter model. Periosteal and endosteal circumferences, cortical area, cortical MC, and toughness were most important among the original features, and periosteal and endosteal circumferences among the BM-normalized features.

In the femoral neck data, the classification accuracy of the LDA models was also quite good although not as high as in the diaphysis data. The 3 lifestyles could be classified with 87.4% cross validation accuracy using the pQCT variables. The BM-normalization had no effect on the overall accuracy, but the aquatic group could be detected slightly better with the original (unnormalized) variables. The most important variables in the unnormalized model were trabecular area, total area, endosteal and periosteal circumferences, and total MC and, in the BM-normalized model, trabecular area, trabecular MD, trabecular MC, and total area. The scatter plots of the data along linear discriminants (Fig. 4c, d) are similar to the diaphysis data, with the well separated aquatic group and some overlap between the other groups. In Fig. 4d, one can also observe an aquatic data point, a *P. hispida botnica* individual, that the LDA model misclassified as semiaquatic. This individual has exceptionally high total and trabecular MDs, reminiscent to semiaquatic animals rather than seals. The neighbouring semiaquatic data point is an *O. zibethicus* individual with normal values for the species. The main reason for the misclassification was probably the high trabecular MD value, since this variable had a very high weight in the BM-normalized model.

With the loading test variables of the femoral neck, only a two-class (semiaquatic, terrestrial) lifestyle classifier could be constructed, since these variables were missing from the seals, yielding 78.8% accuracy with the original features and 81.8% accuracy with the BM-normalized features. In these models, none of the variables had particularly high weights, the most important variables being toughness in the unnormalized model and F_max_ in the normalized model. A species level classifier using all neck pQCT and loading test variables could separate the available 6 species with 81.8% accuracy, if no normalization was used, and with 86.4% accuracy after BM-normalization. Despite good overall accuracy, only 1 species (*O. zibethicus*) was detected perfectly in the first model and none in the latter. The most important features in the unnormalized case were total area, trabecular area, deformation at maximum load (D_max_), cortical area, and periosteal circumference and, in the BM-normalized case, trabecular MD, trabecular area, total MD, total area, periosteal circumference, trabecular MC, and cortical area.

In the last LDA experiment, accurate classifiers were obtained for separating *N. vison* and *M. putorius* using all femoral diaphysis or all femoral neck variables. When the diaphysis data were used, the accuracy was 92.6% both with the original and BM-normalized features. The models were not identical, but most of the important features were the same: cortical MC, cortical area, periosteal and endosteal circumferences, F_max_, stiffness, and cortical MD, accompanied by E_max_ in the unnormalized case and cortical thickness and deformation at yield (D_yield_) in the BM-normalized case.

When the neck data were used, the accuracy with unnormalized features was 81.8% and with normalized features 86.4%. Once again, most of the important features were same in both models: trabecular area, total area, total MC, cortical area, total MD, periosteal circumference, trabecular MC, trabecular MD, E_max_, and D_yield_, accompanied by endosteal circumference and toughness in the unnormalized case and cortical thickness, force at yield, D_max_, and F_max_ in the normalized case. The similarity of the unnormalized and BM-normalized models demonstrated that BM-normalization has only a minimal effect when the species have a similar BM and body shape. On the other hand, the large number of high-weight variables suggests that the classification task was complex, and a combination of many features was needed to separate the 2 species.

### Hierarchical clustering

**Fig. 5:**
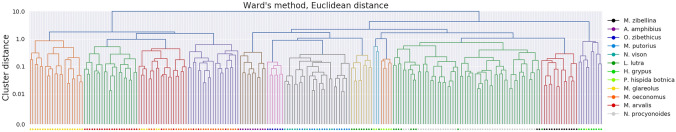
Dendrogram of the optimal clustering of the femoral diaphysis data using species as the reference class (K = 13, NMI = 0.840, all 12 species, n = 232)

The optimal species clustering of the femoral diaphysis data (pQCT and three-point bending variables) was obtained by using 6 features (original cortical MD, cortical area, D_max_, and F_max_, and BM-normalized cortical MC and stiffness), Wardʼs method, and K = 13 (NMI = 0.840). The confusion table (Supplementary Fig. S2) shows that the clusters matched the species quite well and nearly all clusters had only 1 dominant species. The only exception was the mixed cluster of *N. vison* and *M. putorius*. In addition, the clustering inserted about half of *L. lutra* into other speciesʼ clusters, most often to that of *N. procyonoides*. The dendrogram (Fig. 5) has 3 main branches: (1) clusters with small voles, (2) cluster C3 with all *H. grypus* and 1 *P. hispida botnica*, and (3) clusters of other animals. This main division is mostly due to large differences in D_max_ and BM-normalized stiffness. In the first branch, the most distinct clusters are C1 (*M. glareolus*, with low cortical MD and high BM-normalized stiffness) and C2 (mostly *M. oeconomus*, with high cortical MD, cortical area, and F_max_ among voles). On a lower level, C5 (*M. arvalis*) is separated from C6 (mostly *M. oeconomus*) by smaller F_max_ and BM-normalized stiffness. The third branch is divided into 2 sub-branches by the combination of all features. In the first sub-branch, the most distinct clusters are C12 (*A. amphibius*, with lower D_max_ and higher BM-normalized cortical MC and stiffness) and C9 (9 *L. lutra* individuals, with higher cortical area and F_max_). On a lower level, C13 (*O. zibethicus*) is clearly separated from C8 (*N. vison* and *M. putorius*) by higher cortical MD and BM-normalized cortical MC. In the second sub-branch, clusters C4 and C11 clearly differ from others by their higher F_max_ and cortical area. C4 is a kind of an outlier cluster containing 2 *P. hispida botnica* individuals with very high cortical MD and 1 *L. lutra* individual with exceptionally high cortical area, F_max_, and BM-normalized cortical MC for the species, while the normal *P. hispida botnica* individuals belong to C11. The remaining 2 clusters, C7 and C10, differ from each other by cortical area, F_max_, and BM-normalized stiffness. C7 consists of all 50 *N. procyonoides* individuals, as well as 8 *L. lutra* individuals with low cortical MD and high D_max_ and 2 M*. zibellina* individuals with very low D_max_ for the species. C10 matches *M. zibellina*, but there is also 1 small *L. lutra* with exceptionally low cortical MD, low cortical area, and F_max_ for the species.

**Fig. 6:**
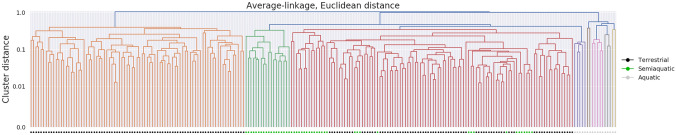
Dendrogram of the optimal clustering of the femoral diaphysis data using lifestyle as the reference class (K = 8, NMI = 0.494, all 12 species, n = 232)

An optimal clustering with the same features but using lifestyle as the reference class was obtained with the average link metric and K = 8 (NMI = 0.494). The confusion table (Supplementary Fig. S3) shows that the aquatic seals were perfectly separated from the other lifestyles, but separation between the terrestrial and semiaquatic mammals was less successful. The 3 main branches of the dendrogram (Fig. 6) separate all small voles and most of the seals from other animals. The second main branch is divided into 3 clusters, corresponding to semiaquatic rodents (with high cortical MD), a large cluster of other medium-sized animals (*L. lutra*, *N. vison*, *M. putorius*, *M. zibellina*, *N. procyonoides*), and a group of 5 *P. hispida botnica* individuals, whose cortical area and F_max_ are very low compared to other seals, being more reminiscent of *L. lutra*. The third main branch covers 4 clusters of seals, 2 of which are quite distinct: a cluster of 2 *P. hispida botnica* individuals with very high cortical MD and another cluster of 2 *H. grypus* individuals with very high D_max_. The clustering hierarchy indicates that individual variation between seals is large (they fall into multiple clusters, despite the same lifestyle), even larger than between medium-sized animal species (*M. zibellina*, *N. vison*, *M. putorius*, *L. lutra*, and *N. procyonoides* fall into the same cluster, despite different lifestyle classes).

**Fig. 7:**
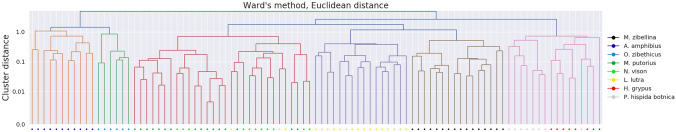
Dendrogram of the optimal clustering of the femoral neck data using species as the reference class (K = 6, NMI = 0.869, 8 species excluding *Myodes* and *Microtus* voles and *N. procyonoides*, n = 95)

The optimal species clustering of the femoral neck data (pQCT variables only) was obtained by using 5 features (original cortical area and trabecular MD, and BM-normalized cortical area, trabecular MC, and periosteal circumference), Ward’s method, and K = 6 (NMI = 0.869). The clusters matched the species even better than in the femoral diaphysis data, which is partially explained by the smaller number of species. The confusion table (Supplementary Fig. S4) shows pure one-species clusters of *A. amphibius*, *M. zibellina*, *O. zibethicus*, and *L. lutra*, and all seals in 1 cluster. There is only 1 mixed cluster of *N. vison*, *M. putorius*, and a few *L. lutra*. The dendrogram (Fig. 7) shows that the main division is between semiaquatic rodents (with very high trabecular MD and BM-normalized trabecular MC) and other animals. The first branch (semiaquatic rodents) is divided into 2 distinct clusters (C1 and C2) matching *A. amphibius* and *O. zibethicus* (the best separating feature being BM-normalized periosteal circumference). The second branch (other animals) falls into 3 distinct sub-branches: C3 cluster of seals (with high cortical area and low BM-normalized cortical area and trabecular MC), C4 cluster of *N. vison*, *M. putorius*, and a few *L. lutra*, and the branch of C5–6 matching *M. zibellina* and *L. lutra* (with the highest trabecular MDs in the second branch). Cluster C5 (*M. zibellina*) differs from C6 (*L. lutra*) by smaller cortical area and higher BM-normalized periosteal circumference and trabecular MC. The 3 *L. lutra* individuals in C4 have very low trabecular MD and cortical area for the species, being more reminiscent of *N. vison* and *M. putorius.*

**Fig. 8:**
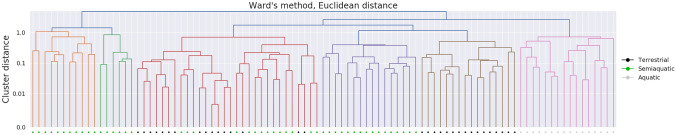
Dendrogram of the optimal clustering of the femoral neck data using lifestyle as the reference class (K = 6, NMI = 0.609, 8 species excluding *Myodes* and *Microtus* voles and *N. procyonoides*, n = 95)

The same optimal clustering was obtained when NMI was evaluated using lifestyle as a reference class (NMI = 0.609). The dendrogram (Fig. 8) structure is also identical, with 5 pure clusters and 1 mixed cluster of semiaquatic and terrestrial species (confusion table in Supplementary Fig. S5). The identical clustering hierarchy in both cases indicates that (1) differences between *A. amphibius* and *O. zibethicus* are pronounced (they fall into different clusters despite having the same lifestyle class), (2) differences between *N. vison*, *M. putorius*, and the few distinct *L. lutra* individuals are minimal (they fall into the same cluster despite different species and lifestyle classes), and (3) differences between *H. grypus* and *P. hispida botnica* are small (they fall into the same cluster despite different species classes).

## Discussion

### Main findings

The aim of the present study was to discover potential relationships between locomotor habits and femoral characteristics in selected mammalian species, including detailed biomechanical data as a methodological novelty in comparative experiments. The main findings were as follows: (1) the aquatic seals did not display increased femoral bone MDs, however, (2) the semiaquatic mammals showed high MD values suggesting osteosclerosis. (3) While none of the individual osteological features could separate species or lifestyles adequately, combinations of multiple features produced very accurate classifiers and clusterings. (4) It seems that various combinations of bone characteristics could provide animals with adequate adaptations to their environment and that they need not to be similar among species in the same niche.

We observed that the multivariate models could show clear groupings of individuals into species and ecological niches even when individual features were inadequate. The LDA classifiers produced very good or even excellent cross validation accuracy, especially from the femoral diaphysis data (Fig. 4). In most models, BM-normalization either improved the accuracy or had no effect, but the very best model (species classification from the diaphysis data), yielding nearly 97% accuracy, used the original features without BM-normalization. Analysis of the classification errors by the diaphysis species classifier reveals that BM-normalization increased to some extent misclassifications between similar species (*N. vison vs. M. putorius*; *H. grypus vs. P. hispida botnica*), but it also generated a few other errors for some individuals, whose BM-normalized variable values deviated from the other members of the species. Interestingly, BM-normalization had the opposite effect in the diaphysis lifestyle classifier, where it decreased misclassifications among *N. vison* and *M. putorius.* Before BM-normalization, most of *M. putorius* (and all *N. vison*) were classified as semiaquatic, probably due to their large size (being among the largest terrestrials in this modelling task), but the BM-corrected model assigned most of *M. putorius* into the terrestrial class, together with 1 misclassified *N. vison*. The special experiments for classifying only *M. putorius* and *N. vison* revealed that the BM-normalization had no (diaphysis) or, at most, minimal (femoral neck) effect, but the classification task was complex, and multiple features were needed to create linear class boundaries.

The clustering results, using an optimal subset of both original and BM-normalized features, were consistent with the LDA. Both femoral diaphysis and neck data produced very good clusterings that reflected well the species division, excluding the confusion between *N. vison* and *M. putorius* and some mislocated *L. lutra* (Figs. 5, 7). The optimal clusterings with respect to lifestyle were less successful, due to the mixed clusters of semiaquatic and terrestrial species (Figs. 6, 8).

Interestingly, the most important features in different models were often the same or at least equivalent (*i.e.*, features with extremely strong linear correlation), independent of whether the class was lifestyle or species. In the original (unnormalized) diaphysis data, the most important original features included cortical area, cortical MC, periosteal and endosteal circumferences, and toughness. Due to strong linear correlations, there is redundancy, and another algorithm might have emphasized only a subset of these features. In the BM-normalized diaphysis data, the species classifier emphasized periosteal and endosteal circumferences, while the lifestyle classifier emphasized cortical area and cortical MC, but BM-normalized cortical area and periosteal circumference were practically equivalent due to strong linear correlation. The best features in clustering are not fully comparable with the LDA features due to slightly different dataset (the goal was to maximize the number of species, which reduced available features), but still the same or equivalent features occurred also there: cortical area, F_max_ (practically equivalent to cortical MC), BM-normalized cortical MC, and BM-normalized stiffness (practically equivalent to BM-normalized periosteal circumference).

In the femoral neck data, the lifestyle and species classifiers were constructed from slightly different data but, yet, there was strong overlap among the most important features: trabecular area, total area, and periosteal circumference in the unnormalized data and trabecular MD, trabecular area, trabecular MC, and total area in the normalized data. Trabecular MD and BM-normalized trabecular MC also occurred among the best clustering features as well as BM-normalized cortical area (practically equivalent to BM-normalized trabecular area).

In summary, HC based on species produced clear separation, while the analyses based on lifestyle were less successful indicating that, especially considering terrestrial and semiaquatic niches, different combinations of osteological features can yield adaptations with individual and evolutionary fitness. The less successful separation by lifestyle could also be due to the restrictions in the availability of species for sampling causing biases in the material.

One of the original questions of this study was to assess how phylogeny and ecology are reflected in the osteological variables. Due to this, we chose both carnivores and rodents to examine, if the classification of the samples was more likely to be based on the niche or the clade. It is, however, important to remember the effect of body size as, for example, *L. lutra* and *N. procyonoides* were of a relatively similar BM and categorized in a mixed manner in the dendrogram displaying femoral diaphysis (Fig. 5). In contrast to smaller rodents, *A. amphibius* and *O. zibethicus* were placed with carnivores in the first division of the dendrogram. Regarding femoral neck (Fig. 7), small voles were excluded due to technical limitations, and the first division clearly separated larger rodents from carnivores. When examining the niche-based dendrograms (Figs. 6, 8), the aquatic seals were clustered together, but the other specimens were mixed. We can suggest that as the semiaquatic species chosen for the study display extensive use of the terrestrial habitat, this would become a determining factor for their osteological properties. In addition, our preliminary data suggest a tentative hypothesis that convergent evolution towards a niche could determine the osteological properties more than the clade does. This would not be unexpected, as convergent evolution into the aquatic niche is a well-known phenomenon described in, for instance, chondrichthyans, ichthyosaurs, cetaceans, and sirenians (McGhee 2011). Interesting comparisons for future studies would include, *e.g.*, the marine iguana (*Amblyrhynchus cristatus*) and the flightless cormorant (*Nannopterum harrisi*) compared to the muskrat and water vole, penguins (Spheniscidae), and pinnipeds but, similar to the present study, species availability and conservation will necessarily restrict many of these interesting prospects.

## Phocids

Phocids (true seals) display a limited capacity of terrestrial locomotion even though they regularly leave the water for extended periods of time for resting, moulting, and breeding (Reidenberg 2007). On land, phocids do not use their extremities to support weight but propel their body forward through the progression of a dorsoventral body wave. Sometimes they are considered semiaquatic rather than true aquatic species that would include sirenians and cetaceans. During aquatic locomotion of phocids, the hind limbs are the major source of propulsion, supplemented by the lateral undulations of the lumbosacral region (Berta et al. 2006). Similar to aquatic taxa, the phocid femur has decreased in size and it is typically short and of anterior-posteriorly flattened shape (Berta et al. 2006; Hafed et al. 2021). When the morphological, cross-sectional, and biomechanical properties of the femurs were compared to the terrestrial species of the present study, the seals were noted to have higher absolute values in most parameters. However, this was not observed for bone MDs that were unexpectedly low compared to the smaller species (Supplementary Table S3, S4). Elevated levels of trabecular MD that reflect the body’s mineral storage would have been in line with earlier literature proposing that the osteological adaptations of aquatic tetrapods include increased bone MD (Wall 1983; Fish and Stein 1991; Gray et al. 2007; Houssaye 2009). It is considered an adaptation to overcome buoyancy during swimming and diving and involves cortical thickening, loss of medullary cavity, and compaction of trabecular bone, but the extent of these modifications could also depend on the diving depth or hydrodynamics determined by, *e.g.*, swimming speed. Previous literature on the osteological characteristics of pinnipeds, especially phocids, is complex (Nakajima and Endo 2013) and, according to Houssaye (2009), pachyostosis is absent in pinnipeds, while osteosclerosis has been observed for the extinct *Valenictus* odobenid. On the other hand, Koretsky and Rahmat (2017) documented pachyosteosclerotic bones in extinct and modern phocids, including the ringed seal.

Phocids have also been reported to reduce their bone mass by periosteal bone resorption, by enlargement of projections with cancellous internal structures, and possibly by reducing trabecular density (Nakajima and Endo 2013), giving support to the results of the present study. The secondarily reduced bone density of some pinnipeds, such as northern elephant seal (*M. angustirostris*) and walrus (*Odobenus rosmarus*), could be associated with lung collapse during deep dives (Wall 1983). Despite the above, the concept of altered bone density in pinnipeds remains obscure, and there are no generally recognized threshold values to objectively determine, whether particular bones display osteosclerosis or pachyostosis (Houssaye et al. 2016). While the aquatic environment gives the animal buoyancy, the moderately high density of water (1.0 g/cm^3^) also subjects limbs to higher mechanical loads during swimming (Kilbourne and Hutchinson 2019). Even though increased bone density due to aquatic niche was not observed in the seals of the present study, strength was still visible in the generally high biomechanical values of the phocids compared to the other studied species. As the femurs are not load-bearing on land but the animals haul-out on their abdomens, bone strength is unlikely to depend on the time spent on land/ice. The importance of the aquatic lifestyle is emphasized by the allometric equations between femoral mass and BM (Supplementary Fig. S1B). In the terrestrial species, femoral mass did show positive allometry with BM, similar to previous data (Prange et al. 1979). However, in the aquatic seals, the allometry was negative and the semiaquatic species occupied an intermediate position. In addition to the aquatic lifestyle, our findings probably reflect the higher body size of the seals. When the mass-corrected data were compared to the studied terrestrial species, the seals had lower pQCT and mechanical strength values (see also Sornay-Rendu et al. 2013 for women), which was probably due to overcorrection (Supplementary Table S7, S8, S9). Moreover, bone morphology explains biomechanics, and the flat femur behaves differently from the bones of the other species, when it is examined with the three-point bending test, and this may be one explanation to the observed higher values in the seals.

There were some interesting differences in the densitometric properties of the femurs between *H. grypus* and *P. hispida botnica*. As the average BM of *H. grypus* was clearly higher (94 *vs.* 33 kg), we expected to find elevated density values in this species, too. However, their cortical and trabecular MDs were similar or lower than those of *P. hispida botnica*. It remains unclear whether these findings could relate to species-specific differences in the amount of time spent in water or in the respective diving behaviors (Thompson et al. 1991; Harkonen et al. 2008; Oksanen et al. 2015). Neither species uses their femurs for support while hauling-out, but both require them for similar activity while foraging. However, *P. hispida* uses its front flipper claws for opening and maintaining breathing holes in the ice (Smith and Hammill 1981), which would also entail simultaneous active use of hind flippers. This could lead to increased requirements of femur strength, but this explanation remains hypothetical. Another supposition relates to the contamination with persistent organic pollutants of the Baltic Sea environment (Sonne et al. 2020), as discussed below.

**Fig. 9:**
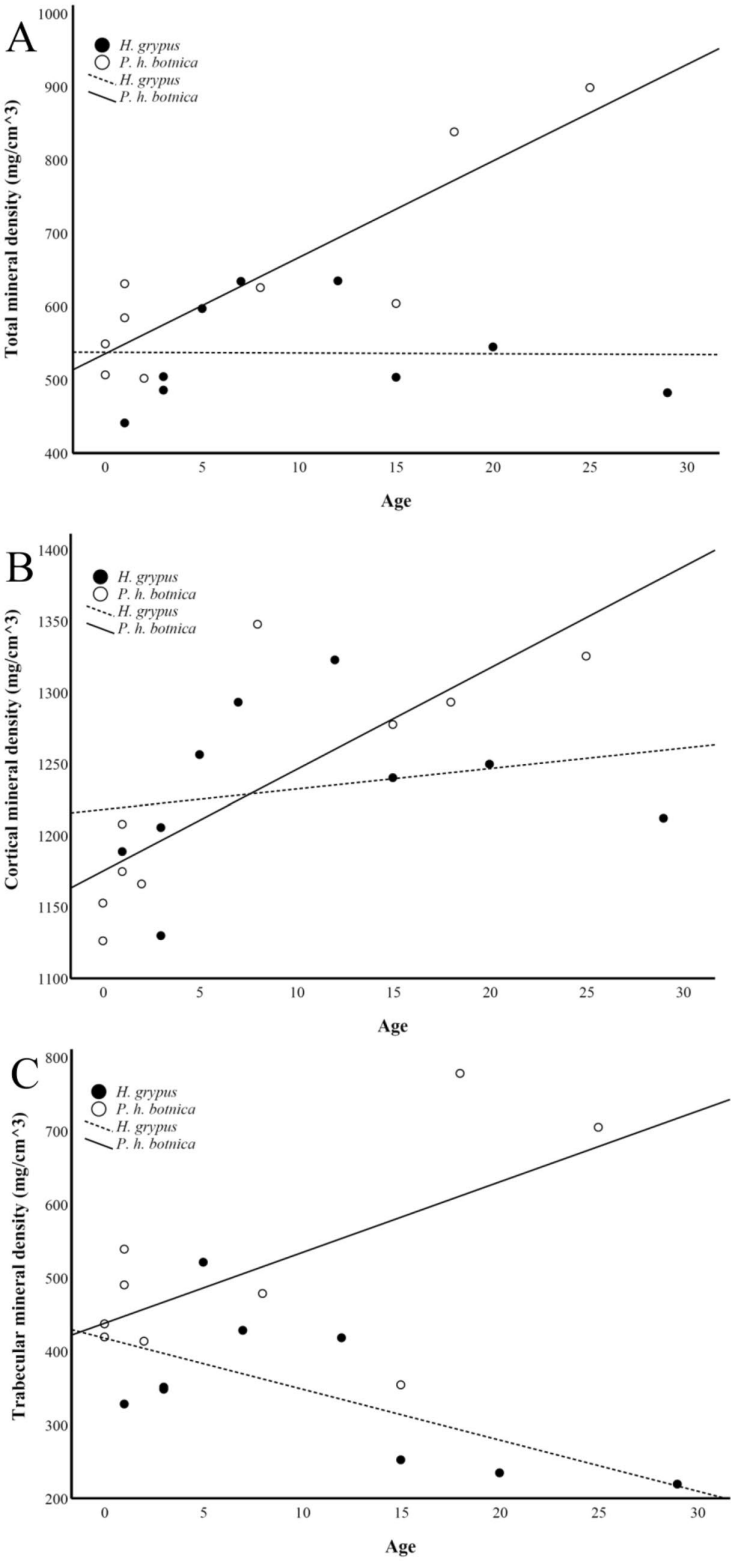
Correlations between age and **a** total mineral density in femoral neck, **b** cortical mineral density in femoral diaphysis, and **c** trabecular mineral density in femoral neck in the Baltic grey and ringed seals. Black symbols = grey seals, white symbols = ringed seals. All correlations were statistically nonsignificant (NS) for the grey seal data. For the ringed seals, total mineral density: r_s_ = 0.706, *p* = 0.034; cortical mineral density: r_s_ = 0.840, *p* = 0.005; trabecular mineral density: NS

Bone density can increase with age in terrestrial and aquatic mammals (Wall 1983; Kahle et al. 2019), but the interspecific variation may presumably be large enough to mask this. In humans, there is an age-related decrease in femoral bone MD from young adulthood onward (Mazess et al. 1990). Regarding the seals examined in the present study, the total and cortical MD values correlated positively with age in *P. hispida botnica*, but this could not be observed for *H. grypus* (Fig. 9a–c). Previously, skull bone lesions, asymmetry, and decreased trabecular MD in radius have been documented in seals during the period of high organochlorine contamination in the Baltic biota (Zakharov and Yablokov 1990; Bergman et al. 1992; Lind et al. 2003). Routti et al. (2008) suggested that bone lesions may be associated with contaminant‐mediated vitamin D and thyroid disruption in *H. grypus*. They observed that the correlations between circulating 1,25-dihydroxyvitamin D_3_, thyroid hormones, and hepatic contaminants were less prominent in *P. hispida botnica*. The results of Routti et al. (2008) are convoluted by the observation that the Baltic seals did not suffer from vitamin D_3_ deficiency, in fact, their hepatic D_3_ levels were higher than those of the reference seals. It can, however, be speculated that the dissociation of femoral bone MD from age of *H. grypus* in the present study could be associated with contaminants via depressed 1,25-dihydroxyvitamin D_3_ levels and/or hyperthyroidism causing bone loss in older individuals, but the issue remains unresolved until further studies.

### Semiaquatic species

**Fig. 10:**
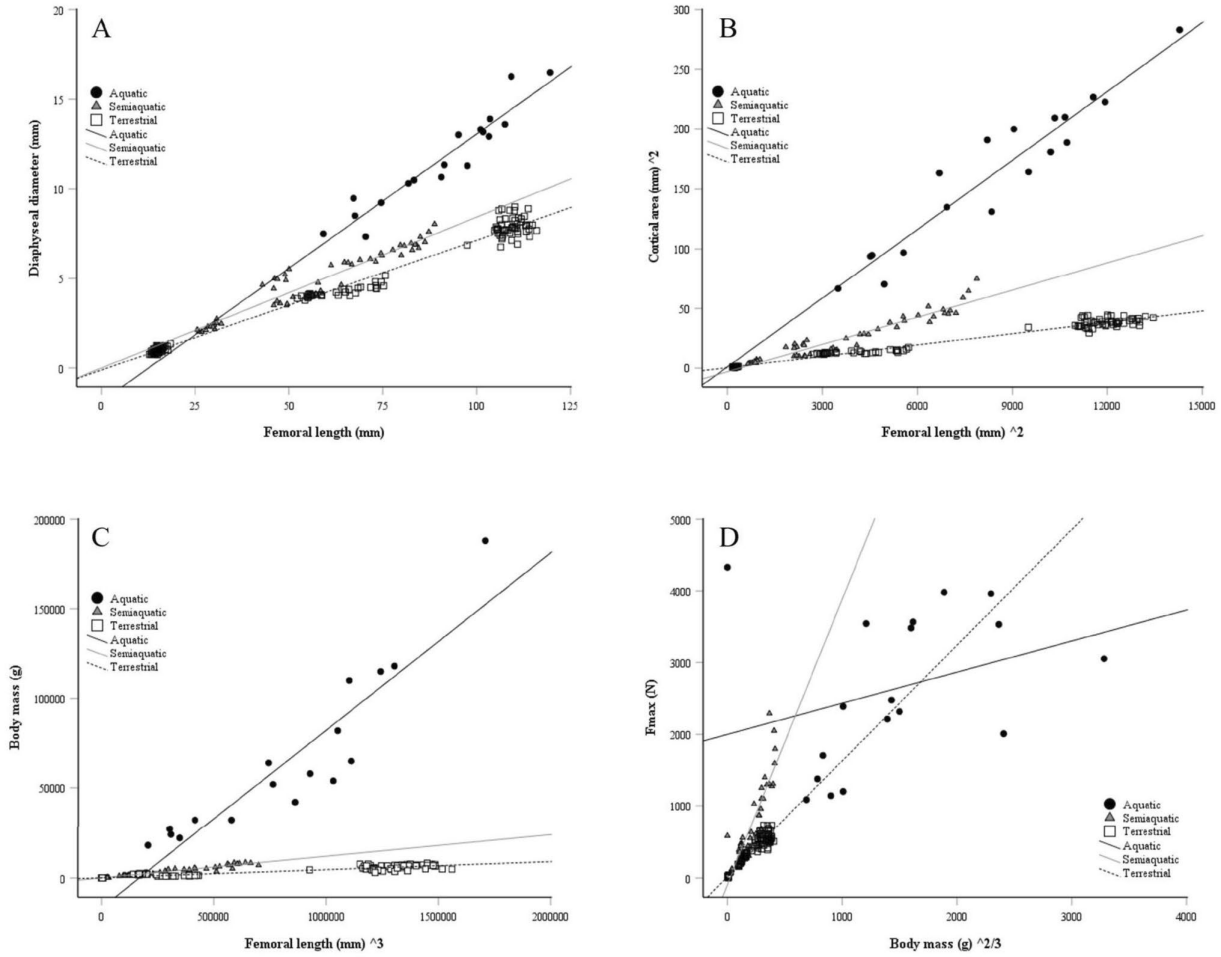
Relationships between the diameter of femoral diaphysis, femoral length, cortical area, body mass, and force at maximum load (F_max_) in aquatic, semiaquatic, and terrestrial mammals. **a** aquatic: r_s_ = 0.955, *p* < 0.001; semiaquatic: r_s_ = 0.949, *p* < 0.001; terrestrial: r_s_ = 0.926, *p* < 0.001; **b** aquatic: r_s_ = 0.926, *p* < 0.001; semiaquatic: r_s_ = 0.948, *p* < 0.001; terrestrial: r_s_ = 0.960, *p* < 0.001; **c** aquatic: r_s_ = 0.953, *p* < 0.001; semiaquatic: r_s_ = 0.980, *p* < 0.001; terrestrial: r_s_ = 0.933, *p* < 0.001; **d** aquatic: r_s_ = 0.420, *p* = 0.082; semiaquatic: r_s_ = 0.907, *p* < 0.001; terrestrial: r_s_ = 0.919, *p* < 0.001

Semiaquatic mammals usually display less distinct osteological modifications in their limbs than aquatic species do (Stein 1989). This probably indicates an evolutionary compromise related to the need to actively locomote in two vastly different environments. The American beaver (*Castor canadensis*) may be one exception with clearly increased deposition of compact bone in its limbs (Stein 1989) and increased cortical thickness can be observed in several otter species (Houssaye and Botton-Divet 2018). The pooled data from the species of the present study showed that the semiaquatic mammals had higher femoral densities compared to the mammals of the other ecological niches, which could indicate, for instance, osteosclerosis. In addition, the diaphyseal stiffness was higher in the semiaquatic carnivores and rodents compared to their terrestrial counterparts within each order. However, the semiaquatic and terrestrial species also showed some similarities, supporting the earlier notion by Currey (2002) that the force required to break a bone when it is loaded axially, in bending, or in torsion would be proportional to BM^2/3^. This phenomenon was observed for the diaphyses of the semiaquatic and terrestrial animals, while the correlation did not reach significance in the aquatic seals, possibly due to the lower number of specimens (Fig. 10d). It must also be recalled that, in the current study, the species had dissimilar body shapes. In all 3 ecological groups, diaphyseal diameter, cortical area, as well as F_max_ showed positive allometry with femoral length (Supplementary Fig. S1C–E). This is understandable, as bones need to become relatively thicker and stronger as they become longer to withstand the challenges of locomotion on land but, interestingly, this also took place in the aquatic environment.

It could be speculated that the higher bone MD values of the semiaquatic species may result from their somewhat higher BMs compared to the terrestrial specimens. However, cortical MD correlated positively with BM in the aquatic and terrestrial species but, in the semiaquatic group, this correlation did not reach significance. This suggests that, in the whole study material, body size would not be an adequate explanation to the differences in cortical MD, but both locomotor habit and size could have influenced our results (see also Kilbourne and Hutchinson 2019 for mustelids). Lower mineralization in the terrestrial habitat could be compatible with the decreased risk of fracture. Regarding the aquatic seals, the morphological adaptations to their ecological niche are considerably more advanced than those of the semiaquatic species, which show significantly less adaptive characteristics in the locomotor, vascular, and respiratory systems. Due to this, we suggest that in the absence of whole-body aquatic morphology, the semiaquatic species would need special osteological adaptations to counteract buoyancy, and this would be detectable in their bone MD and related variables.

Semiaquatic rodents have relatively robust bones, short femurs, enlarged muscular attachments, and elongated hind feet compared to other rodents (Samuels and Van Valkenburgh 2008). In the present study, the semiaquatic *O. zibethicus* had high cortical and trabecular MD values in femurs, dissimilar to earlier literature that was based on different methodology (Stein 1989). *O. zibethicus* is primarily a surface swimmer and shallow diver with propulsive appendages that act as paddles to produce thrust, and it can stay submerged for up to 20 min (Willner et al. 1980; Fish 2000). It has non-wettable fur that provides positive buoyancy increasing the energetic costs of diving (Fish et al. 2002). Thus, the elevated bone MD would help it to increase the specific gravity to overcome buoyancy. The present study also observed high trabecular MD values for *A. amphibius*, whereas Stein (1989) reported low bone densities for this species. According to Samuels and Van Valkenburgh (2008), *A. amphibius* may lack pronounced skeletal specializations due to its mutually conflicting adaptations to both aquatic and fossorial habits. *Microtus* voles were previously documented to have dense limb bones without an apparent thickening of the cortical layer (Stein 1989), but the present study found low cortical MD, cross-sectional, and mechanical values for the small voles, while most BM-corrected values were the highest among all species. A possible technical issue that could result in the overestimation of MD in rodents is the resolution limit of pQCT regarding small bones. On the other hand, the partial volume effect can also be a source of error, *i.e.*, if the bone is very small compared to resolution, it is possible to underestimate its density (Brodt et al. 2003).

Overall, the ability of swimming and shallow diving does not seem to involve extensive osteological modifications in the limbs (Stein 1989). This is supported by the results of Martín-Serra et al. (2014), who found that the shape of hind limb bones of carnivores, including terrestrial, arboreal, and semiaquatic species among others, was strongly influenced by body size and phylogeny but less so by locomotor behavior. As long as terrestrial locomotion is an important part of the life strategy of a species, it is necessary to maintain adequate bone strength to support the BM of an individual. At the same time, semiaquatic animals have to avoid allocating too much energy to diving, which would be caused by light bones and excessive buoyancy. Hence, the balance of these demands may be reflected in the complex findings of bone characteristics, especially between the semiaquatic and terrestrial species.

### Mustelids

Mustelids are interesting study subjects for morpho-functional research, as this clade has undergone several evolutionary transitions of lifestyle and includes fossorial/digging specialists (*e.g.*, badgers), natatorial/swimming specialists (otters, mink), scansorial/climbing specialists (marten, sable), and more generalized species (polecats) (Amson and Kilbourne 2019). According to Fish and Stein (1991), mustelids displayed an increasing trend in limb bone densities from terrestrial to aquatic species. In addition, a greater bone volume/total volume fraction in the femoral and humeral heads was found in natatorial taxa (Amson and Kilbourne 2019), and natatorial and scansorial species had the highest and lowest values of limb cross-sectional traits, respectively (Kilbourne and Hutchinson 2019; Parsi-Pour and Kilbourne 2020).

In the present study, 4 mustelid species with an average BM ranging from 1.2 kg (*M. zibellina*) to 5.9 kg (*L. lutra*) were examined and, for most of the measured parameters, *L. lutra* had the highest absolute values. This could be partly due to the semiaquatic lifestyle and a potential ballast role for long bones (Botton-Divet et al. 2016, 2017). Descent may not be the determining factor here, as the clearly land-dwelling species of similar size (*N. procyonoides*) had more similar bone properties to otters than close mustelid relatives did, suggesting that the higher body size would be the most likely determinant for bone properties (Fish and Stein 1991). According to Kilbourne and Hutchinson (2019), the front limb bones of otters have greater relative resistance to compression and bending and greater structural strength than those of mustelids with other locomotor habits but, in this case, the different use of the otter’s front limbs, especially in manipulating prey, could also be of importance. Generally, our findings of high F_max_, stiffness, and toughness in the femoral diaphysis of *L. lutra* (Supplementary Table S5) support this earlier literature. The species displays efficient terrestrial locomotion and spends only 12–18% of its time hunting in water (Nolet and Kruuk 1989; Beja 1996). It is still much more specialized to the aquatic habitat than *N. vison* that has mostly similar osteological values to *M. putorius* and *M. zibellina*, both of which are considered mainly terrestrial, while the latter is also a good climber (Larivière and Jennings 2009). Compared to *L. lutra*, *N. vison* lacks specialized appendages for the aquatic lifestyle and uses a quadrupedal paddling style that is energetically expensive (Williams 1983). Accordingly, there can be a marked divergence in the long bone morphology between Mustelinae and Lutrinae species (Botton-Divet et al. 2016).

Even though *N. vison* mostly displayed similar densitometric, cross-sectional, and biomechanical values to *M. putorius* in the present study, the LDA on the diaphyseal variables separated these species with over 90% cross validation accuracy. This suggests that while individual osteological variables did not clearly differ between these species of a similar body size and shape, the combination of these features may indicate adaptation to ecological niche. The higher trabecular MD of *N. vison* could derive from a need for buoyancy control (Fish and Stein 1991). On the other hand, *M. zibellina* that is more scansorial in nature than the other studied mustelids (Amson and Kilbourne 2019) had surprisingly high absolute values of, for instance, total and trabecular MDs and trabecular MC of the femoral neck (Supplementary Table S4). These may be related to the larger size of its femur compared to those of *N. vison* and *M. putorius* despite the lower BM. When compared to the other medium-sized mustelids studied, *M. zibellina* also had longer femurs, lower cortical MD, stiffness, and F_max_, but higher toughness of the diaphysis. Some of these features could relate to the scansorial lifestyle, preventing the breaking of bones in the case the animal falls from a tree. According to Kilbourne (2017), scansorial mustelids have relatively elongated and gracile bones compared to those with other locomotor specializations. These could be advantageous adaptations enabling better navigation in their climbing habitats. When the measured variables were size-corrected by BM, *M. zibellina* had the highest values for several pQCT and biomechanical parameters among mustelids.

### Limitations

There are some limitations in the present study to be considered. Obviously, the selection of taxa was not optimal and the great differences in the BMs of the animals became an issue, but the constraints by availability dictated the choice of the species for the analyses. Due to the relatively low number of available species, the effects of common descent on bone properties could not be thoroughly assessed. The physical activity levels of the laboratory- and farm-bred animals were necessarily lower than in nature, which could have affected the bone MD and related variables (Mages et al. 2021). In addition, the body size of farm-bred carnivores is usually higher than that of their wild counterparts (Mustonen et al. 2009). The age structure differed between species, and specimens of both sexes were not available for all of them, limiting the detection of intraspecific variation.

Regarding methodology, it is possible that the pQCT method includes some cortical bone when calculating the trabecular parameters of rodents due to the small size of their femurs. It is also important to mention that due to the resolution of pQCT instruments, bone MD reflects more of the volumetric density including porosity that can be different from the true density of the bone tissue itself. Furthermore, the aspect ratio for the seals was not optimal for the bending test.

Concerning data analyses, determination of important features from LDA models is incomplete due to strong correlations between variables. While features with high loadings do have a strong effect on classification, the modelling algorithm emphasizes only some of the strongly correlated features and other equally important features may remain unrecognized. Because of the relatively large variation in osteological properties caused, for instance, by different age of the animals, HC also revealed within-species groupings that were indicative of the heterogeneity of the subject material.

However, intraspecific variation was generally very small; for all the diaphysis variables and femoral neck pQCT variables the species-wise standard deviations were significantly smaller than the means (*i.e.*, coefficient of variation was <1). The only exceptions were 2 bending test variables, D_yield_ (high variation in *O. zibethicus*) and E_yield_ (high variation in multiple species), in the femoral neck data. However, most variables were size-dependent and the species-wise means and standard deviations were of different magnitudes. Therefore, individual variation of large animals (seals) may be small when compared to their species-wise means, but high in comparison to other species. This effect is cumulative when distances are calculated over multiple features. A good example is the clustering of the diaphysis data, when lifestyle was used as a reference class: the seals were allocated into multiple clusters despite the same lifestyle (demonstrating large multivariate distances between individuals), while all medium-sized carnivores were grouped to 1 cluster (small multivariate distances) (Fig. 6).

## Conclusions

While the measured osteological parameters did not show perfect classification of the studied mammalian species based on their ecological niches, relatively clear relationships between particular bone characteristics and lifestyles could still be discerned. In the univariate analyses, none of the individual features could separate species or lifestyles adequately, but the combinations of multiple features produced very good or excellent classifications and clusterings. In the seals, the ecological niche and aquatic locomotion seemed to allow for less dense femoral bones than could be expected based on the BM alone. In contrast, the semiaquatic mammals had high average values for bone MDs, suggesting osteosclerosis to counteract buoyancy. The overall morphology of the bones was diverse, and a particular combination of osteological properties that would be shared by all species in a niche did not seem to be crucial in the adaptation to aquatic, semiaquatic, or terrestrial environment.

### Supplementary Information

Below is the link to the electronic supplementary material.

Supplementary file1 (PDF 1064 kb)

## Data Availability

The datasets generated and analyzed during the current study are available from the corresponding author on reasonable request.

## Abbreviations

BM: Body mass
D_max_: Deformation at maximum load
D_yield_: Deformation at yield
E_max_: Energy at maximum load
E_yield_: Energy at yield
F_max_: Force at maximum load
GLM: Generalized linear model
HC: Hierarchical clustering
K: Number of clusters
LDA: Linear discriminant analysis
LOO: Leave-one-out
MC: Mineral content
MD: Mineral density
NMI: Normalized mutual information
pQCT: Peripheral quantitative computed tomography
r_s_: Spearman correlation coefficient
SSE: Sum of squared error

## Declarations

### Code availability

The Python code for hierarchical clustering with dendrogram visualizations is available at https://github.com/saaralehtiniemi/hierarchical-clustering.
