# Supplementary material for: Osteological profiling of femoral diaphysis and neck in aquatic, semiaquatic, and terrestrial carnivores and rodents: effects of body size and locomotor habits
Source: J Comp Physiol B. 2024 Apr 27;194(4):473–92. doi: 10.1007/s00360-024-01551-7 (PMC11316726; doi:10.1007/s00360-024-01551-7)
Supplement: Supplementary file 1 — Supplementary file1 (PDF 1064 kb) [file 360_2024_1551_MOESM1_ESM.pdf]

Nieminen et al. Osteological profiling of femoral diaphysis and neck in aquatic, semiaquatic, and terrestrial carnivores and rodents: effects of body size and locomotor habits

Supplementary Table S1. The resolution of the pQCT imaging.

Species	Pixel dimension x, y, z (mm)
H. grypus	0.689 × 0.689 × 1.250
P. h. botnica	0.689 × 0.689 × 1.250
L. lutra	0.295 × 0.295 × 1.250
N. vison	0.148 × 0.148 × 1.250
M. putorius	0.148 × 0.148 × 1.250
M. zibellina	0.148 × 0.148 × 1.250
N. procyonoides	0.148 × 0.148 × 1.250
O. zibethicus	0.148 × 0.148 × 1.250
A. amphibius	0.148 × 0.148 × 1.250
M. glareolus	0.092 × 0.092 × 1.250
M. oeconomus	0.092 × 0.092 × 1.250
M. arvalis	0.092 × 0.092 × 1.250

Supplementary Table S2. Femoral mass (g, mg in *M. glareolus*), volume (ml), and dimensions (mm), mean ± SE.

**Order**/species/n	Femoral mass	Femoral volume	Femoral length[a]	Femoral length[b]	Diameter of diaphysis[c]	Diameter of neck	Diameter of caput	Distance from trochanter to caput
**Carnivora**								
H. grypus (9)	87.6 ± 6.68	61.8 ± 4.10	91.5 ± 2.41	103.9 ± 2.63	13.6 ± 0.61/27.4 ± 1.07	14.0 ± 0.40	24.2 ± 0.39	57.1 ± 1.17
P. h. botnica (9)	37.2 ± 5.52	25.5 ± 3.84	68.1 ± 3.39	76.6 ± 3.96	9.6 ± 0.59/18.5 ± 1.26	9.6 ± 0.38	18.0 ± 0.38	42.4 ± 2.07
L. lutra (22)	11.7 ± 0.73	7.3 ± 0.42	73.2 ± 1.63	77.5 ± 1.73	6.6 ± 0.13	6.7 ± 0.12	11.5 ± 0.15	23.6 ± 0.47
N. vison (12)	2.5 ± 0.28	1.6 ± 0.18	51.0 ± 1.72	53.7 ± 1.71	4.0 ± 0.13	4.3 ± 0.23	6.4 ± 0.25	15.7 ± 0.68
M. putorius (16)	2.7 ± 0.07	1.7 ± 0.05	52.8 ± 0.65	55.9 ± 0.61	4.0 ± 0.03	5.4 ± 0.07	6.9 ± 0.05	14.9 ± 0.11
M. zibellina (16)	3.1 ± 0.13	1.9 ± 0.08	66.0 ± 1.00	70.0 ± 1.05	4.5 ± 0.07	4.1 ± 0.10	7.0 ± 0.12	14.0 ± 0.20
N. procyonoides (50–51)	16.8 ± 0.23	12.1 ± 0.16	103.3 ± 0.47	109.2 ± 0.48	7.9 ± 0.07	8.0 ± 0.09	12.9 ± 0.07	27.5 ± 0.26
**Rodentia**								
O. zibethicus (7)	3.3 ± 0.19	2.0 ± 0.15	43.0 ± 0.89	47.1 ± 0.93	5.0 ± 0.14	3.4 ± 0.10	7.0 ± 0.13	15.2 ± 0.35
A. amphibius (13)	0.4 ± 0.02	0.3 ± 0.01	27.1 ± 0.55	29.0 ± 0.55	2.3 ± 0.06	1.5 ± 0.04	3.1 ± 0.04	7.4 ± 0.12
M. glareolus (28)	37.5 ± 0.89[d]	≈ 0.05	14.4 ± 0.12	14.8 ± 0.13	1.0 ± 0.02	1.0 ± 0.04	1.4 ± 0.01	3.1 ± 0.05
M. oeconomus (30)	nd	≈ 0.07	14.4 ± 0.41	15.9 ± 0.19	1.1 ± 0.02	1.1 ± 0.01	1.7 ± 0.01	3.5 ± 0.05
M. arvalis (28–29)	nd	≈ 0.03	13.8 ± 0.16	14.5 ± 0.15	0.9 ± 0.02	1.1 ± 0.02	1.5 ± 0.01	3.4 ± 0.08

[a] = *fossa intercondylaris–fossa trochanterica*, [b] = *condyli femoris–caput femoris*, [c] = two measurements given for phocids, as their femurs are flattened along the limb axis, [d] = mg, nd = not determined

Supplementary Table S3. Peripheral quantitative computed tomography characteristics of the femoral diaphysis, mean ± SE.

**Order**/species/n	Cortical mineral density (mg/cm^3)	Cortical mineral content (mg)	Cortical area (mm^2)	Cortical thickness (mm)	Periosteal circumference (mm)	Endosteal circumference (mm)
P Lifestyle	<0.001[a,b]	<0.001[a]	<0.001[a]	<0.001[a,b]	<0.001[a,b]	<0.001[a,b]
P Body mass	<0.001	<0.001	<0.001	<0.001	<0.001	<0.001
P Interaction	<0.001	<0.001	<0.001	<0.001	<0.001	<0.001
**Carnivora**						
H. grypus (9)	1233 ± 19	230 ± 17.0	202 ± 14.3	3.3 ± 0.17	63 ± 3.0	37 ± 2.8
P. h. botnica (9)	1230 ± 27	144 ± 22.3	124 ± 16.9	2.3 ± 0.17	43 ± 2.9	19 ± 2.1
L. lutra (21)	1381 ± 19	58 ± 4.4	43 ± 2.7	1.3 ± 0.03	26 ± 0.7	11 ± 0.2
N. vison (12)	1382 ± 7	17 ± 1.3	13 ± 0.9	0.7 ± 0.03	15 ± 0.5	7 ± 0.4
M. putorius (16)	1378 ± 3	16 ± 0.2	13 ± 0.2	0.7 ± 0.01	14 ± 0.1	6 ± 0.1
M. zibellina (16)	1258 ± 6	17 ± 0.4	14 ± 0.4	0.8 ± 0.04	16 ± 0.2	9 ± 0.2
N. procyonoides (40–51)	1307 ± 6	50 ± 0.8	38 ± 0.5	1.4 ± 0.02	28 ± 0.2	18 ± 0.3
**Rodentia**						
O. zibethicus (7)	1495 ± 11	28 ± 1.2	19 ± 0.9	0.9 ± 0.02	18 ± 0.4	9 ± 0.4
A. amphibius (13)	1344 ± 22	7 ± 0.5	5 ± 0.3	0.4 ± 0.02	9 ± 0.3	3 ± 0.1
M. glareolus (24–28)	973 ± 14	0.8 ± 0.03	1.0 ± 0.02	0.1 ± 0.01	4.1 ± 0.05	2.2 ± 0.04
M. oeconomus (30)	1088 ± 10	1.3 ± 0.05	1.2 ± 0.03	nd	nd	nd
M. arvalis (28)	1033 ± 9	1.0 ± 0.03	0.9 ± 0.03	nd	nd	nd

nd = not determined (see text for details), [a] = statistically significant difference between aquatic and terrestrial species, [b] = statistically significant difference between semiaquatic and terrestrial species (generalized linear model)

Supplementary Table S4. Peripheral quantitative computed tomography characteristics of the femoral neck, mean ± SE.

**Order**/species/n	Cortical area (mm^2)	Cortical thickness (mm)	Periosteal circumference (mm)	Endosteal circumference (mm)	Total mineral density (mg/cm^3)	Total mineral content (mg)	Total area (mm^2)	Trab. mineral density (mg/cm^3)	Trab. mineral content (mg)	Trabecular area (mm^2)
P Lifestyle	<0.001[a]	<0.001[a]	<0.001[a,b]	<0.001[a]	<0.001[a,b]	<0.001[a]	<0.001[a]	<0.001[b]	<0.001[a]	<0.001[a]
P Body mass	0.215	0.039	<0.001	<0.001	0.004	<0.001	<0.001	<0.001	<0.001	<0.001
P Interaction	<0.001	<0.001	<0.001	<0.001	0.008	<0.001	<0.001	<0.001	<0.001	<0.001
**Carnivora**										
H. grypus (8–9)	140 ± 14	2.8 ± 0.21	60 ± 1.3	42 ± 3.0	537 ± 23	158 ± 8	295 ± 11	345 ± 33	45 ± 4.2	133 ± 5.0
P. h. botnica (9)	108 ± 13	2.2 ± 0.17	44 ± 1.9	24 ± 2.4	638 ± 47	102 ± 12	158 ± 14	513 ± 47	36 ± 4.2	71 ± 6.5
L. lutra (21–22)	59 ± 8	1.5 ± 0.16	29 ± 1.8	12 ± 1.4	735 ± 27	53 ± 6.4	74 ± 12	618 ± 25	20 ± 2.3	33 ± 5.6
N. vison (11–12)	13 ± 0.9	0.7 ± 0.08	16 ± 0.8	10 ± 0.9	686 ± 28	14 ± 1.1	21 ± 2.2	502 ± 33	4.6 ± 0.28	10 ± 1.0
M. putorius (15–16)	17 ± 0.6	0.8 ± 0.06	18 ± 0.3	10 ± 0.5	638 ± 11	17 ± 0.5	27 ± 1.0	412 ± 21	5.0 ± 0.25	12 ± 0.4
M. zibellina (16)	17 ± 0.8	0.7 ± 0.05	15 ± 0.3	4 ± 0.6	791 ± 14	16 ± 0.6	21 ± 0.9	706 ± 24	6.5 ± 0.31	9 ± 0.4
N. procyonoides (39–48)	nd	nd	31 ± 0.4	18 ± 0.6	628 ± 12	47 ± 1.1	75 ± 1.6	453 ± 10	15 ± 0.4	34 ± 0.7
**Rodentia**										
O. zibethicus (5–7)	28 ± 3.9	0.8 ± 0.14	19 ± 1.4	1.0 ± 0.25	1026 ± 26	30 ± 3.8	30 ± 3.8	1065 ± 25	14 ± 1.7	13 ± 1.7
A. amphibius (9–13)	5 ± 0.4	0.4 ± 0.04	8 ± 0.4	1.0 ± 0.13	799 ± 19	5 ± 0.3	6.1 ± 0.46	939 ± 33	2.5 ± 0.16	2.7 ± 0.20
M. glareolus	nd	nd	nd	nd	nd	nd	nd	nd	nd	nd
M. oeconomus	nd	nd	nd	nd	nd	nd	nd	nd	nd	nd
M. arvalis	nd	nd	nd	nd	nd	nd	nd	nd	nd	nd

nd = not determined (see text for details), [a] = statistically significant difference between aquatic and terrestrial species, [b] = statistically significant difference between semiaquatic and terrestrial species (generalized linear model)

Supplementary Table S5. Characteristics of the three-point bending of the femoral diaphysis, mean ± SE.

**Order**/species/n	Stiffness (N/mm)	D_{yield} (mm)	F_{yield} (N)	E_{yield} (mJ)	D_{max} (mm)	F_{max} (N)	E_{max} (mJ)	Toughness (mJ)
P Lifestyle	<0.001[a]	<0.001[a,b]	<0.001[a]	<0.001[a]	0.001[a]	<0.001[a]	<0.001[a]	0.272
P Body mass	<0.001	<0.001	<0.001	<0.001	<0.001	<0.001	<0.001	<0.001
P Interaction	<0.001	<0.001	<0.001	<0.001	<0.001	<0.001	<0.001	<0.001
**Carnivora**								
H. grypus (9)	2156 ± 127	0.5 ± 0.03	981 ± 86	239 ± 31	3.3 ± 0.22	3012 ± 259	6663 ± 645	8673 ± 1128
P. h. botnica (9)	2962 ± 545	0.4 ± 0.03	1025 ± 163	191 ± 27	1.7 ± 0.18	2250 ± 413	2713 ± 687	2800 ± 673
L. lutra (21)	1368 ± 146	0.5 ± 0.04	649 ± 70	180 ± 28	1.9 ± 0.11	1162 ± 106	1407 ± 100	1488 ± 94
N. vison (11)	635 ± 74	0.4 ± 0.01	217 ± 20	40 ± 4	1.0 ± 0.05	345 ± 44	255 ± 41	259 ± 41
M. putorius (16)	471 ± 15	0.5 ± 0.01	206 ± 4	50 ± 2	1.3 ± 0.05	318 ± 7	281 ± 14	385 ± 18
M. zibellina (16)	186 ± 11	0.6 ± 0.03	115 ± 7	37 ± 4	2.3 ± 0.09	204 ± 9	343 ± 22	616 ± 48
N. procyonoides (50)	469 ± 14	nd	nd	nd	1.7 ± 0.04	538 ± 13	nd	nd
**Rodentia**								
O. zibethicus (7)	815 ± 65	0.3 ± 0.02	257 ± 13	45 ± 4	1.0 ± 0.06	445 ± 24	272 ± 28	295 ± 36
A. amphibius (11–13)	229 ± 20	0.3 ± 0.01	70 ± 6	11 ± 1.3	0.5 ± 0.03	88 ± 7	24 ± 3.3	31 ± 3.8
M. glareolus (27–28)	176 ± 6	0.1 ± <0.01	14 ± 0.7	0.6 ± 0.05	0.2 ± 0.01	20 ± 0.8	2.0 ± 0.15	3.1 ± 0.26
M. oeconomus (30)	175 ± 6	nd	nd	nd	0.2 ± 0.01	25 ± 1.1	nd	nd
M. arvalis (28)	101 ± 5	nd	nd	nd	0.2 ± 0.01	14 ± 0.6	nd	nd

D = deformation, F = force, E = energy at yield and maximum load, nd = not determined (see text for details), [a] = statistically significant difference between aquatic and terrestrial species, [b] = statistically significant difference between semiaquatic and terrestrial species (generalized linear model)

Supplementary Table S6. Characteristics from the femoral neck loading test, mean ± SE.

**Order**/species/n	Stiffness (N/mm)	D_{yield} (mm)	F_{yield} (N)	E_{yield} (mJ)	D_{max} (mm)	F_{max} (N)	E_{max} (mJ)	Toughness (mJ)
P Lifestyle	<0.001[b]	0.710	0.047[b]	0.438	0.785	<0.001[b]	0.157	0.036[b]
P Body mass	<0.001	0.067	0.012	0.080	<0.001	<0.001	0.023	0.007
P Interaction	0.221	0.433	0.212	0.332	0.142	<0.001	0.060	0.009
**Carnivora**								
H. grypus	nd	nd	nd	nd	nd	nd	nd	nd
P. h. botnica	nd	nd	nd	nd	nd	nd	nd	nd
L. lutra (17–20)	168 ± 23	2.0 ± 0.27	305 ± 54	368 ± 98	5.5 ± 0.44	660 ± 74	1808 ± 239	2111 ± 232
N. vison (11–12)	410 ± 29	0.3 ± 0.05	103 ± 17	18 ± 6	1.7 ± 0.10	291 ± 17	311 ± 25	332 ± 21
M. putorius (16)	434 ± 31	0.4 ± 0.09	142 ± 22	45 ± 16	1.5 ± 0.08	343 ± 10	314 ± 20	324 ± 20
M. zibellina (16)	478 ± 33	0.3 ± 0.06	132 ± 16	28 ± 11	1.3 ± 0.13	329 ± 20	287 ± 35	318 ± 40
N. procyonoides (50)	292 ± 12	nd	nd	nd	4.4 ± 0.20	734 ± 23	nd	nd
**Rodentia**								
O. zibethicus (6)	420 ± 50	0.2 ± 0.11	76 ± 22	16 ± 12	1.4 ± 0.22	234 ± 19	194 ± 42	195 ± 43
A. amphibius (12–13)	239 ± 21	0.1 ± 0.01	31 ± 2	2.4 ± 0.23	0.4 ± 0.03	64 ± 6	16 ± 2.4	15 ± 2.3
M. glareolus (25)	96 ± 6	nd	nd	nd	0.3 ± 0.03	16 ± 0.6	nd	nd
M. oeconomus (30)	nd	nd	nd	nd	0.4 ± 0.02	23 ± 0.8	nd	nd
M. arvalis (28)	nd	nd	nd	nd	0.3 ± 0.02	18 ± 0.7	nd	nd

D = deformation, F = force, E = energy at yield and maximum load, nd = not determined (see text for details), [b] = statistically significant difference between semiaquatic and terrestrial species (generalized linear model)

Supplementary Table S7. Body mass-normalized characteristics from peripheral quantitative computed tomography of the femoral diaphysis, mean ± SE.

**Order**/species/n	Cortical mineral content (mg/g)	Cortical area (mm^2/g)	Cortical thickness (mm/g)	Periosteal circumference (mm/g)	Endosteal circumference (mm/g)
P Lifestyle	<0.001[a]	<0.001[a]	<0.001[a]	<0.001[a]	<0.001[a]
**Carnivora**					
H. grypus (9)	0.003 ± 0.0002	0.002 ± 0.0002	0.00004 ± 0.000004	0.0008 ± 0.00008	0.0005 ± 0.00005
P. h. botnica (8)	0.004 ± 0.0003	0.004 ± 0.0002	0.00007 ± 0.000005	0.001 ± 0.0001	0.0007 ± 0.00008
L. lutra (20)	0.010 ± 0.0005	0.008 ± 0.0004	0.0003 ± 0.00002	0.005 ± 0.0003	0.002 ± 0.0002
N. vison (12)	0.009 ± 0.0004	0.007 ± 0.0003	0.0004 ± 0.00003	0.008 ± 0.0005	0.004 ± 0.0003
M. putorius (16)	0.008 ± 0.0002	0.006 ± 0.0002	0.0004 ± 0.00001	0.007 ± 0.0002	0.003 ± <0.0001
M. zibellina (16)	0.014 ± 0.0003	0.012 ± 0.0003	0.0007 ± 0.00004	0.014 ± 0.0004	0.008 ± 0.0003
N. procyonoides (40–51)	0.009 ± 0.0003	0.007 ± 0.0002	0.0003 ± <0.00001	0.005 ± 0.0001	0.003 ± <0.0001
**Rodentia**					
O. zibethicus (7)	0.02 ± 0.001	0.02 ± <0.001	0.0007 ± 0.00002	0.01 ± <0.001	0.007 ± 0.0003
A. amphibius (13)	0.03 ± 0.001	0.03 ± 0.001	0.002 ± 0.0001	0.04 ± 0.002	0.02 ± 0.001
M. glareolus (24–28)	0.04 ± <0.001	0.04 ± <0.001	0.006 ± 0.0004	0.18 ± 0.004	0.10 ± 0.003
M. oeconomus (30)	0.05 ± 0.001	0.05 ± 0.001	nd	nd	nd
M. arvalis (28–29)	0.04 ± 0.002	0.04 ± 0.001	nd	nd	nd

nd = not determined (see text for details), [a] = statistically significant difference between aquatic and terrestrial species (generalized linear model)

Supplementary Table S8. Body mass-normalized characteristics from peripheral quantitative computed tomography of the femoral neck, mean ± SE.

**Order**/species/n	Cortical area (mm^2/g)	Cortical thickness (mm/g)	Periosteal circumference (mm/g)	Endosteal circumference (mm/g)	Total mineral content (mg/g)	Total area (mm^2/g)	Trab. mineral content (mg/g)	Trabecular area (mm^2/g)
P Lifestyle	<0.001[a]	<0.001[a]	<0.001[a,b]	<0.001[a]	<0.001[a,b]	<0.001[a,b]	<0.001[a,b]	<0.001[a,b]
**Carnivora**								
H. grypus (8–9)	0.002 ± 0.0003	0.00004 ± 0.000006	0.0008 ± 0.00010	0.0005 ± 0.00009	0.002 ± 0.0002	0.004 ± 0.0005	0.001 ± 0.0001	0.002 ± 0.0002
P. h. botnica (8)	0.003 ± 0.0003	0.00007 ± 0.000009	0.002 ± 0.0002	0.0009 ± 0.00012	0.003 ± 0.0002	0.005 ± 0.0005	0.001 ± 0.0001	0.002 ± 0.0002
L. lutra (20–21)	0.010 ± 0.0009	0.0003 ± 0.00002	0.005 ± 0.0003	0.002 ± 0.0003	0.009 ± 0.0007	0.013 ± 0.0015	0.003 ± 0.0003	0.006 ± 0.0007
N. vison (11–12)	0.007 ± 0.0004	0.0004 ± 0.00003	0.008 ± 0.0005	0.005 ± 0.0004	0.007 ± 0.0003	0.010 ± 0.0006	0.002 ± 0.0002	0.005 ± 0.0003
M. putorius (15–16)	0.008 ± 0.0002	0.0004 ± 0.00003	0.009 ± 0.0002	0.005 ± 0.0003	0.009 ± 0.0003	0.014 ± 0.0006	0.002 ± 0.0001	0.006 ± 0.0003
M. zibellina (16)	0.015 ± 0.0005	0.0006 ± 0.00005	0.013 ± 0.0003	0.004 ± 0.0005	0.014 ± 0.0004	0.018 ± 0.0006	0.006 ± 0.0003	0.008 ± 0.0002
N. procyonoides (39–48)	nd	nd	0.005 ± 0.0002	0.003 ± 0.0001	0.008 ± 0.0003	0.013 ± 0.0005	0.003 ± 0.0001	0.006 ± 0.0002
**Rodentia**								
O. zibethicus (5–7)	0.02 ± 0.003	0.0006 ± 0.00011	0.014 ± 0.0010	0.0008 ± 0.00023	0.023 ± 0.002	0.02 ± 0.003	0.011 ± 0.0011	0.010 ± 0.0011
A. amphibius (9–13)	0.03 ± 0.002	0.002 ± 0.0002	0.04 ± 0.002	0.005 ± 0.0008	0.024 ± 0.001	0.03 ± 0.002	0.012 ± 0.0006	0.014 ± 0.0009
M. glareolus	nd	nd	nd	nd	nd	nd	nd	nd
M. oeconomus	nd	nd	nd	nd	nd	nd	nd	nd
M. arvalis	nd	nd	nd	nd	nd	nd	nd	nd

nd = not determined (see text for details), [a] = statistically significant difference between aquatic and terrestrial species, [b] = statistically significant difference between semiaquatic and terrestrial species (generalized linear model)

Supplementary Table S9. Body mass-normalized characteristics from the three-point bending of the femoral diaphysis, mean ± SE.

**Order**/species/n	Stiffness (N/mm/g)	D_{yield} (mm/g)	F_{yield} (N/g)	E_{yield} (mJ/g)	D_{max} (mm/g)	F_{max} (N/g)	E_{max} (mJ/g)	Toughness (mJ/g)
P Lifestyle	<0.001[a,b]	<0.001[a]	<0.001[a]	<0.001[a]	<0.001[a]	<0.001[a,b]	<0.001[a,b]	<0.001[a]
**Carnivora**								
H. grypus (9)	0.03 ± 0.003	0.00001 ± 0.000001	0.01 ± 0.002	0.003 ± 0.0005	0.00004 ± 0.000004	0.04 ± 0.004	0.08 ± 0.007	0.10 ± 0.012
P. h. botnica (8)	0.08 ± 0.006	0.00001 ± 0.000003	0.03 ± 0.003	0.006 ± 0.0010	0.00006 ± 0.000007	0.06 ± 0.006	0.07 ± 0.015	0.08 ± 0.014
L. lutra (20)	0.24 ± 0.017	0.0001 ± 0.00001	0.11 ± 0.008	0.03 ± 0.004	0.0004 ± 0.00005	0.20 ± 0.010	0.25 ± 0.017	0.27 ± 0.017
N. vison (11)	0.32 ± 0.015	0.0002 ± 0.00002	0.11 ± 0.006	0.02 ± 0.002	0.0006 ± 0.00005	0.17 ± 0.009	0.12 ± 0.011	0.13 ± 0.011
M. putorius (16)	0.23 ± 0.009	0.0002 ± 0.00001	0.10 ± 0.003	0.02 ± 0.001	0.0006 ± 0.00003	0.16 ± 0.004	0.14 ± 0.007	0.19 ± 0.008
M. zibellina (16)	0.16 ± 0.004	0.0006 ± 0.00003	0.10 ± 0.003	0.03 ± 0.002	0.002 ± 0.0001	0.17 ± 0.004	0.29 ± 0.016	0.52 ± 0.028
N. procyonoides (50)	0.08 ± 0.003	nd	nd	nd	0.0003 ± 0.00001	0.09 ± 0.003	nd	nd
**Rodentia**								
O. zibethicus (7)	0.63 ± 0.032	0.0003 ± 0.00003	0.20 ± 0.007	0.04 ± 0.003	0.0008 ± 0.00006	0.35 ± 0.007	0.21 ± 0.019	0.23 ± 0.024
A. amphibius (11–13)	1.15 ± 0.096	0.002 ± 0.0001	0.36 ± 0.022	0.06 ± 0.005	0.002 ± 0.0001	0.44 ± 0.017	0.12 ± 0.009	0.15 ± 0.013
M. glareolus (27–28)	7.64 ± 0.254	0.003 ± 0.0002	0.59 ± 0.029	0.03 ± 0.002	0.007 ± 0.0004	0.87 ± 0.032	0.09 ± 0.006	0.13 ± 0.011
M. oeconomus (30)	7.09 ± 0.191	nd	nd	nd	0.007 ± 0.0003	1.01 ± 0.026	nd	nd
M. arvalis (28)	4.72 ± 0.210	nd	nd	nd	0.008 ± 0.0005	0.65 ± 0.030	nd	nd

D = deformation, F = force, E = energy at yield and maximum load, nd = not determined (see text for details), [a] = statistically significant difference between aquatic and terrestrial species, [b] = statistically significant difference between semiaquatic and terrestrial species (generalized linear model)

Supplementary Table S10. Body mass-normalized characteristics from the femoral neck loading test, mean ± SE.

**Order**/species/n	Stiffness (N/mm/g)	D_{yield} (mm/g)	F_{yield} (N/g)	E_{yield} (mJ/g)	D_{max} (mm/g)	F_{max} (N/g)	E_{max} (mJ/g)	Toughness (mJ/g)
P Lifestyle	0.001[b]	0.096	0.257	0.481	<0.001[b]	0.155	0.574	0.649
**Carnivora**								
H. grypus	nd	nd	nd	nd	nd	nd	nd	nd
P. h. botnica	nd	nd	nd	nd	nd	nd	nd	nd
L. lutra (16–19)	0.03 ± 0.003	0.0004 ± 0.00009	0.05 ± 0.006	0.065 ± 0.0155	0.0012 ± 0.00018	0.12 ± 0.008	0.34 ± 0.035	0.37 ± 0.029
N. vison (11–12)	0.23 ± 0.029	0.0001 ± 0.00002	0.05 ± 0.006	0.008 ± 0.0021	0.0009 ± 0.00010	0.15 ± 0.010	0.16 ± 0.015	0.17 ± 0.014
M. putorius (16)	0.22 ± 0.015	0.0002 ± 0.00004	0.07 ± 0.011	0.022 ± 0.0078	0.0008 ± 0.00004	0.17 ± 0.005	0.16 ± 0.010	0.16 ± 0.009
M. zibellina (16)	0.42 ± 0.034	0.0003 ± 0.00005	0.11 ± 0.012	0.023 ± 0.0082	0.0011 ± 0.00012	0.28 ± 0.018	0.25 ± 0.032	0.28 ± 0.037
N. procyonoides (50)	0.05 ± 0.002	nd	nd	nd	0.0008 ± 0.00004	0.13 ± 0.004	nd	nd
**Rodentia**								
O. zibethicus (6)	0.35 ± 0.055	0.0002 ± 0.00008	0.06 ± 0.017	0.013 ± 0.0093	0.0011 ± 0.00017	0.19 ± 0.010	0.16 ± 0.032	0.16 ± 0.032
A. amphibius (12–13)	1.21 ± 0.120	0.0008 ± 0.00008	0.16 ± 0.016	0.013 ± 0.0015	0.0022 ± 0.00017	0.32 ± 0.029	0.08 ± 0.010	0.08 ± 0.010
M. glareolus (25)	4.23 ± 0.274	nd	nd	nd	0.013 ± 0.001	0.69 ± 0.027	nd	nd
M. oeconomus (30)	nd	nd	nd	nd	0.017 ± 0.0009	0.93 ± 0.022	nd	nd
M. arvalis (28)	nd	nd	nd	nd	0.016 ± 0.001	0.84 ± 0.028	nd	nd

D = deformation, F = force, E = energy at yield and maximum load, nd = not determined (see text for details), [b] = statistically significant difference between semiaquatic and terrestrial species (generalized linear model)

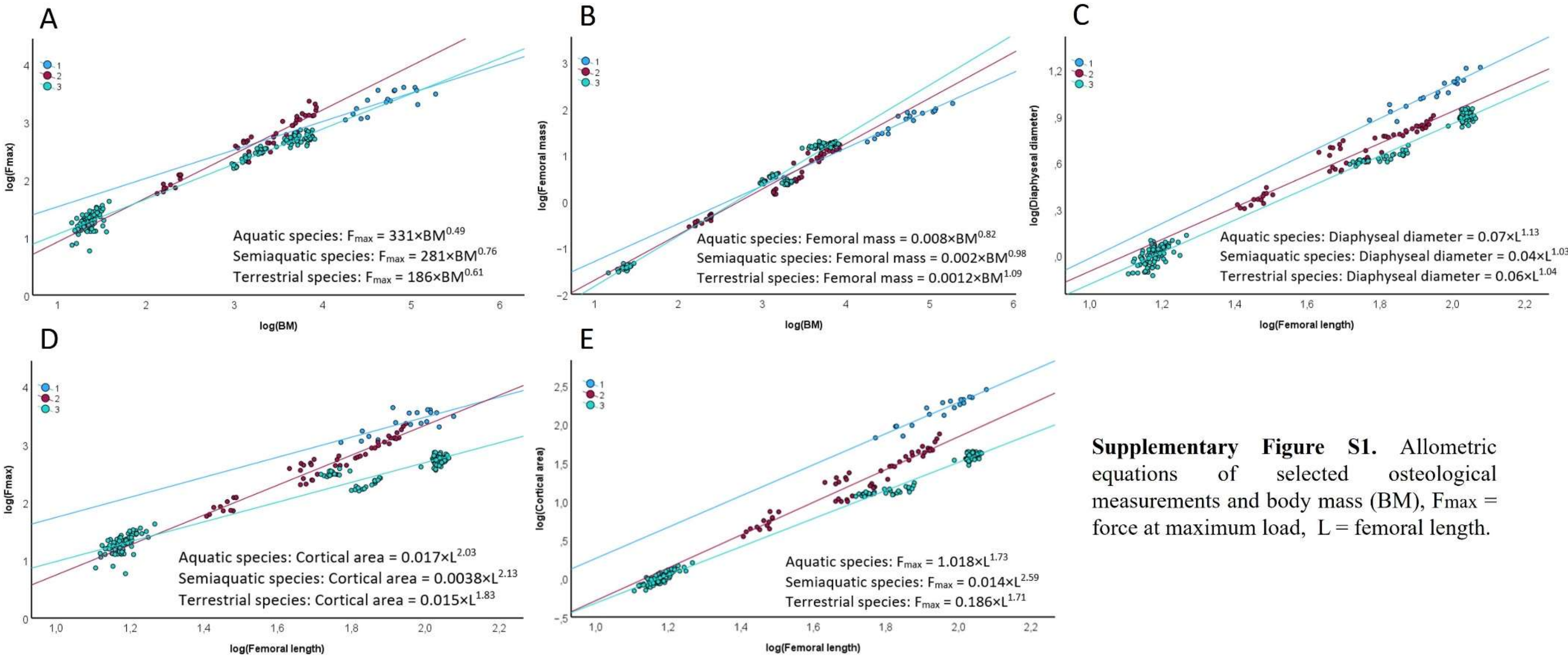

Supplementary Figure S1. Allometric equations of selected osteological measurements and body mass (BM), F_{max} = force at maximum load, L = femoral length.

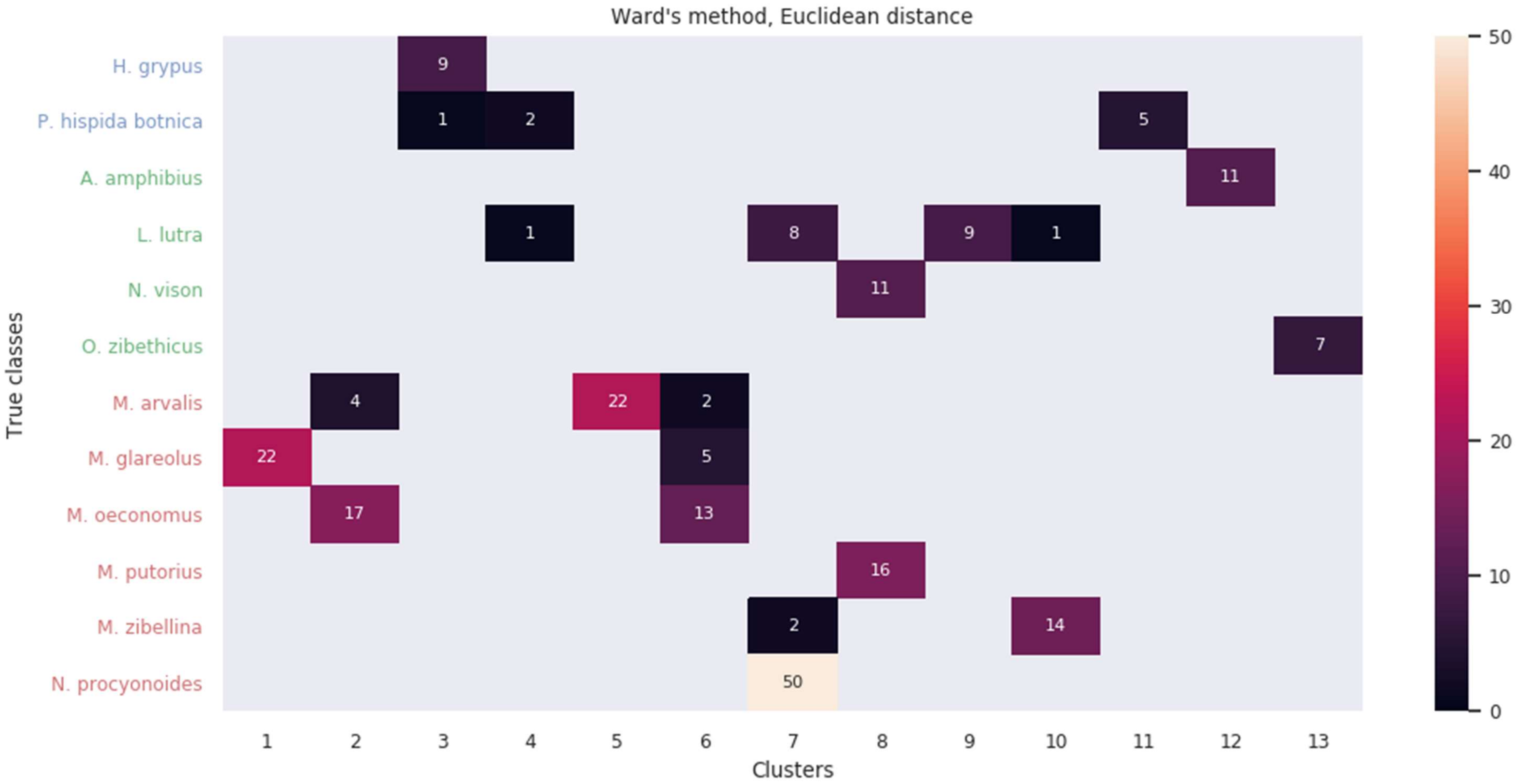

Supplementary Figure S2. Confusion table between species and clusters of the optimal clustering of the femoral diaphysis data.

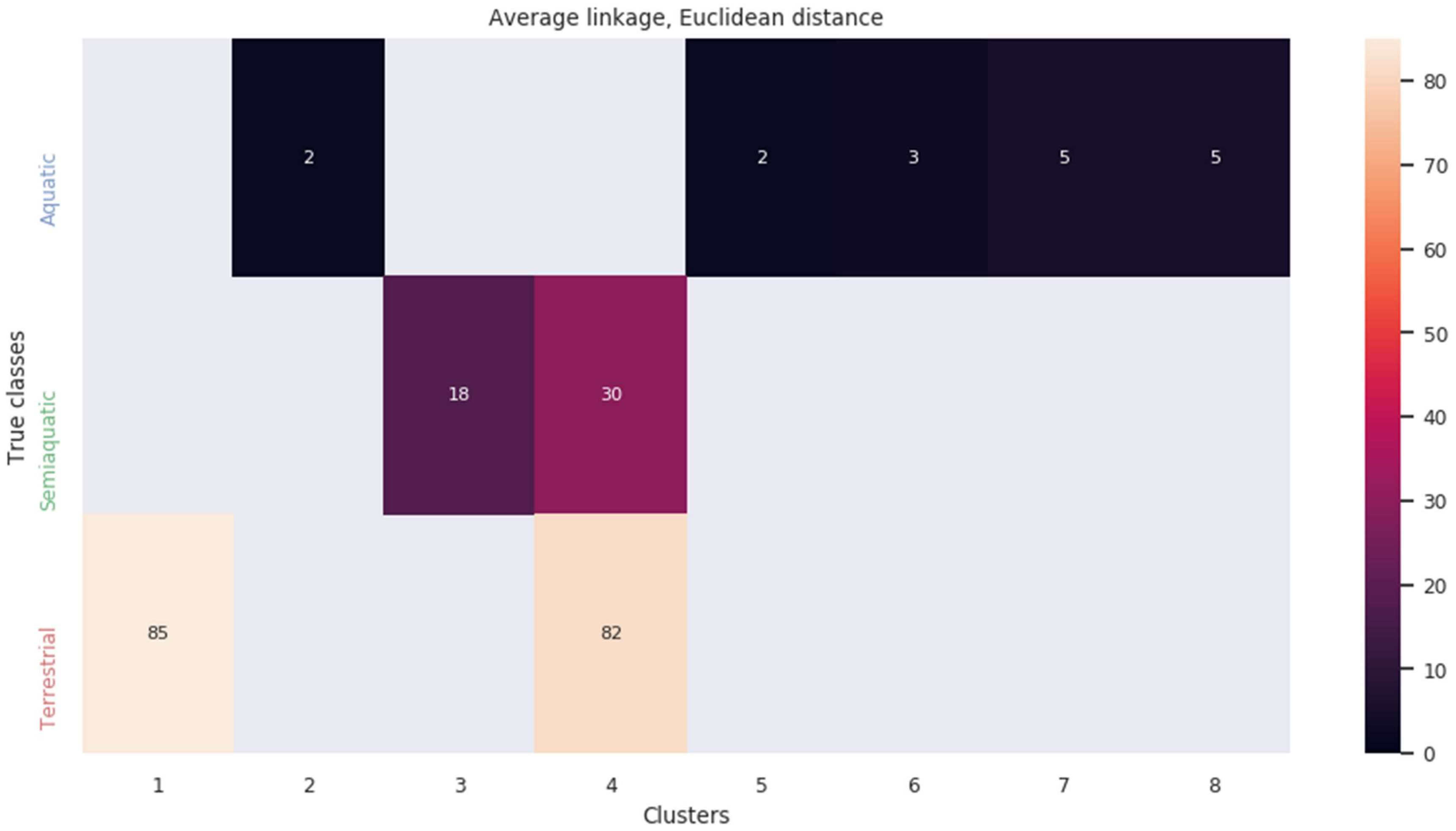

Supplementary Figure S3. Confusion table between lifestyles and clusters of the optimal clustering of the femoral diaphysis data.

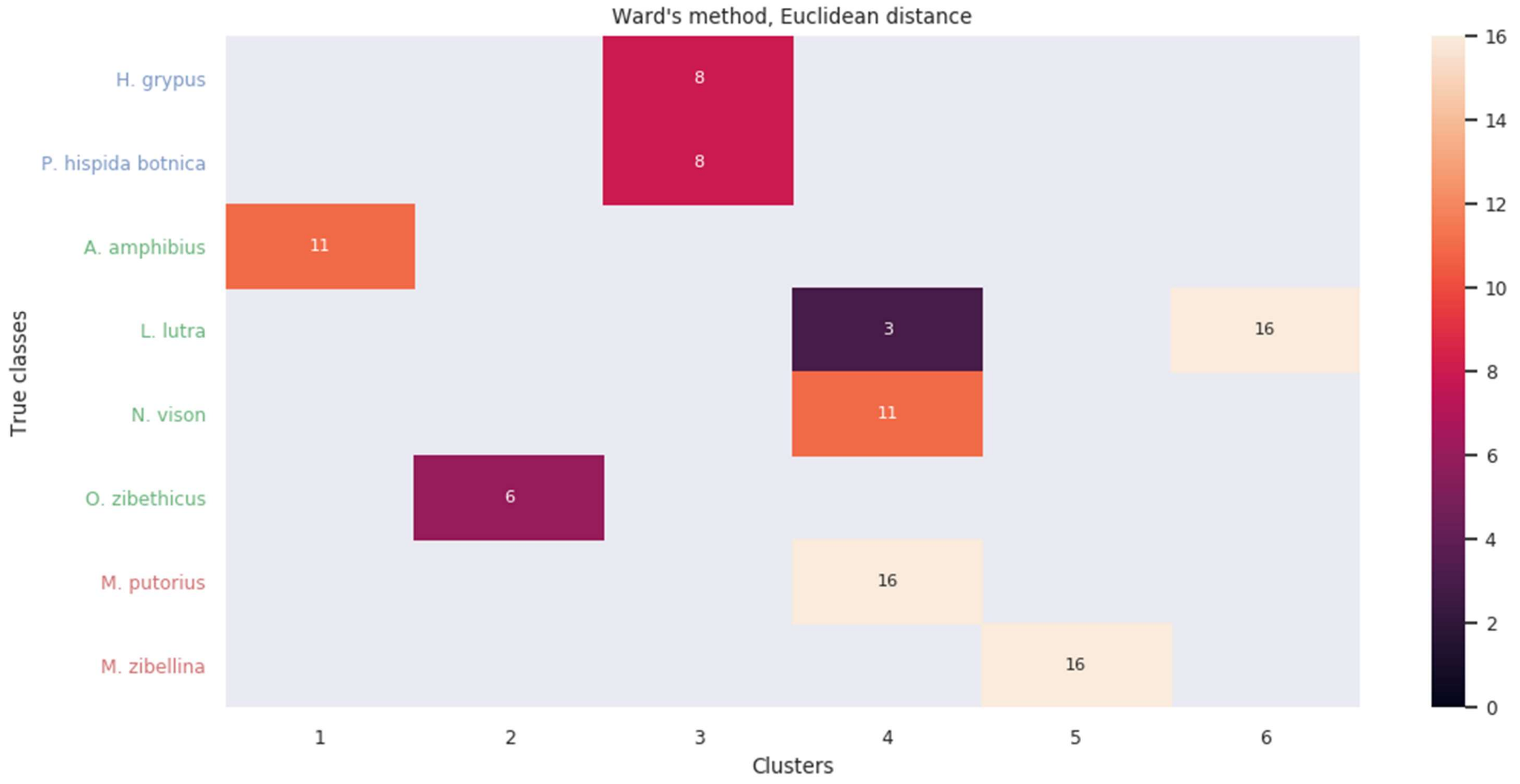

Supplementary Figure S4. Confusion table between species and clusters of the optimal clustering of the femoral neck data.

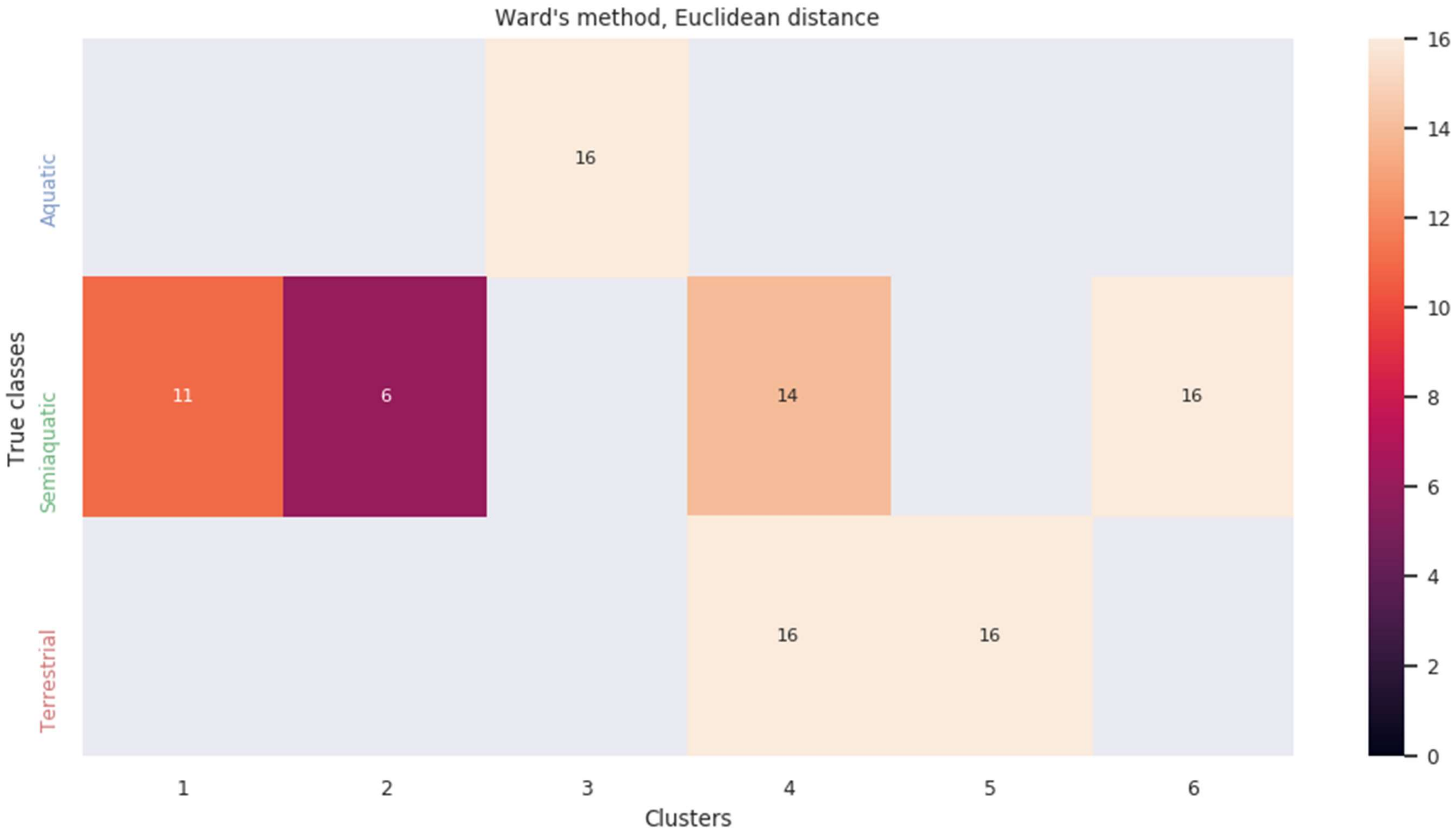

Supplementary Figure S5. Confusion table between lifestyles and clusters of the optimal clustering of the femoral neck data.
